# Ecological risk assessment of predicted marine invasions in the Canadian Arctic

**DOI:** 10.1371/journal.pone.0211815

**Published:** 2019-02-07

**Authors:** Jesica Goldsmit, Christopher McKindsey, Philippe Archambault, Kimberly L. Howland

**Affiliations:** 1 Fisheries and Oceans Canada, Maurice Lamontagne Institute, Mont-Joli, Quebec, Canada; 2 Department of Biology, Science and Engineering Faculty, Laval University, Quebec City, Quebec, Canada; 3 Fisheries and Oceans Canada, Arctic Research Division, Freshwater Institute, Winnipeg, Manitoba, Canada; University of Waikato, NEW ZEALAND

## Abstract

Climate change is impacting environmental conditions, especially with respect to temperature and ice cover in high latitude regions. Predictive models and risk assessment are key tools for understanding potential changes associated with such impacts on coastal regions. In this study relative ecological risk assessment was done for future potential introductions of three species in the Canadian Arctic: periwinkle *Littorina littorea*, soft shell clam *Mya arenaria* and red king crab *Paralithodes camtschaticus*. These species occur in locations connected to Canadian Arctic ports through shipping and have the potential to be introduced via ballast water discharge. The methodology proposed in this study is unique in the sense that it considers not only ballast water origin, but also the distribution of the species being assessed and the sensitivity of the receiving habitat. It combines detailed information (ballast water source of each tank, transit time, time of the year when the water is released, environmental suitability of receiving habitat, impact, and habitat sensitivity) in order to assess ecological risk. Through the use of this approach it is highlighted that domestic discharge events pose a higher relative overall risk on a vessel-specific and cumulative annual bases than international discharges. The main ports of Deception Bay and Churchill were classified as being at moderate to high relative risk for *L*. *littorea* and *M*. *arenaria*, especially from domestic vessels, while relative overall risk for *P*. *camtschaticus* was low for international vessels and null for domestic vessels due to few ships transiting from its range of distribution to Canadian Arctic ports. This work can serve as an approach to help build a list of potential high risk species–a “grey” watch list–for the Canadian Arctic, and provides useful information for consideration in future decision making actions such as the identification of high risk pathways, species and ports.

## Introduction

Invasive species and global warming are among the most serious drivers of global environmental change and threaten marine biodiversity [1–3]. Successful establishment of an invasive species depends upon it completing a series of transitions, each with independent probabilities of failure [4, 5]. Vectors must uptake, transport, and deliver a sufficient number of viable propagules to an area outside of the species’ historic range. These individuals must be capable of surviving, reproducing, and establishing under ambient physico-chemical and biological-ecological conditions [6].

The principal global vector for unintentional introductions of aquatic non-indigenous species (NIS) is shipping [3, 4, 7]. Species may be transported unintentionally during ballast water uptake/discharge and through the accumulation and transport of organisms on vessel surfaces (biofouling), including hulls and protected areas, such as sea chests [8, 9]. The global shipping network is responsible for approximately 90% of global trade [10, 11], posing a substantial concern as it is the dominant vector of aquatic introductions. The “path length” between any two ports is the minimum number of connections or steps required to travel between them (based on recorded voyages in a given year) [10]. Most source-arrival destination pairs are connected by two or less steps, with a maximum of eight [10]. This global connectivity highlights the importance of understanding shipping pathways to evaluate risks associated with NIS since, once established, it is rarely possible to eliminate them [12].

Commercial shipping has thus been estimated to have contributed between 44% to 78% of primary invasions of all NIS to North America [13]. Mid-ocean ballast water exchange (BWE) has been the primary means of reducing the risks of introducing NIS by transoceanic vessels [14]. Water and associated aquatic organisms from coastal ports in ballast tanks of merchant vessels can be effectively replaced with oceanic water and species through BWE (e.g., 97–99% efficiency) [15]. This not only removes a great proportion of all organisms taken up with ballast in source ports but also helps reduce invasion risk by some organisms due to the salinity shock encountered by individuals remaining in tanks following BWE. Although this method has been shown to be very effective for freshwater species [16], its efficacy for coastal marine species is variable [14] and may even increase invasion risk if novel (to receiving ports) oceanic species are taken up during BWE [17].

Sea surface temperature in the Arctic is warming faster than in other parts of the globe [18]. It is predicted that by 2070 a complete open water season will last half of the year [19]. Although predicted changes may sound extreme they may in fact be conservative given that current reductions in sea ice are happening faster than model predictions [20]. Seasonal minimal sea ice extent is estimated to be declining at a rate of 12.4% per decade [21, 22]. Minimum sea ice extent records are increasingly being broken, consistent with the inclusion of anthropogenic forcing in climate modeling [23]. To date, most introductions have occurred in warmer, temperate regions, where there is greater shipping activity [24]. However, the Arctic is increasingly at risk of introductions due to global warming, resource exploitation, increased project developments, and the associated increased shipping activity [25–28]. Indeed, Arctic shipping traffic has increased over the last few years [29] and is expected to raise the risk of NIS introductions to Arctic waters [30–32]. This trend is expected to continue with new shipping routes predicted to open across the Arctic (e.g., the Northwest Passage linking the Atlantic and Pacific oceans through the Canadian Arctic) by mid-century [25, 28].

As most reported marine NIS are benthic [33], it is of particular interest to evaluate the potential for these organisms to be introduced to the Canadian Arctic. Recent studies have demonstrated that potentially NIS benthic species are being transported to the region [34, 35]. To date, three potential shipping-mediated NIS have been described–the red alga *Dumontia contorta*, the tunicate *Polycarpa pomaria*, and the tube-dwelling crustacean *Monocorophium insidosium* [36, 37]. In addition, there are species that have recently been identified as cryptogenic (species that could be either native or non-native) [36–38]. In contrast to the Canadian Arctic, numerous NIS and novel species have recently been reported from other high-latitude areas [35, 39–47]. However, the Canadian Arctic benthic community has generally been poorly sampled because of its size and remoteness, with few systematic surveys having been conducted, making the detection of newly established species difficult [48–50]. Given that high-latitude regions are predicted to experience a significant number of NIS introductions [31, 32, 44], we can only project how these changes could affect the region.

In cases where information is scarce, risk assessments can serve as an effective tool to estimate risk potential in a systematic way [51]. A risk assessment is the process by which undesired events (e.g., NIS introduction and impact) are identified and their consequences parameterized, including uncertainties related to the assessment process [52]. These types of studies can be used to evaluate the invasion potential associated with different shipping pathways and management strategies [53]. Species-level risk assessments provide information about the particular risk of a given species and risk is calculated with direct consideration of the characteristics of the organism [54]. Performing assessments that predict the risk of potential invasion and impact of a given species in regions where species have not yet arrived and/or established can be useful to identify and prevent undesirable future impacts [55, 56]. The development and use of watch lists, combined with monitoring efforts in regions where these types of assessments have been done, can lead to the discovery of NIS before they negatively impact the ecosystem [57, 58].

The aim of the present study is to characterize the relative ecological risk of potential future NIS introductions in Canadian Arctic ports, with special emphasis on the development of a species-specific assessment protocol. The proposed methodology is a unique combination of risk components that allows for comparative analyses between species and shipping pathways being assessed, and ports that have the potential to receive their propagules through ballast water discharge. This risk assessment framework provides information to support management decisions regarding the development of preventive actions to limit new introductions and serves as a starting point to build a list of species with potential risk for the Canadian Arctic.

## Materials and methods

### Study area

**Fig 1:**
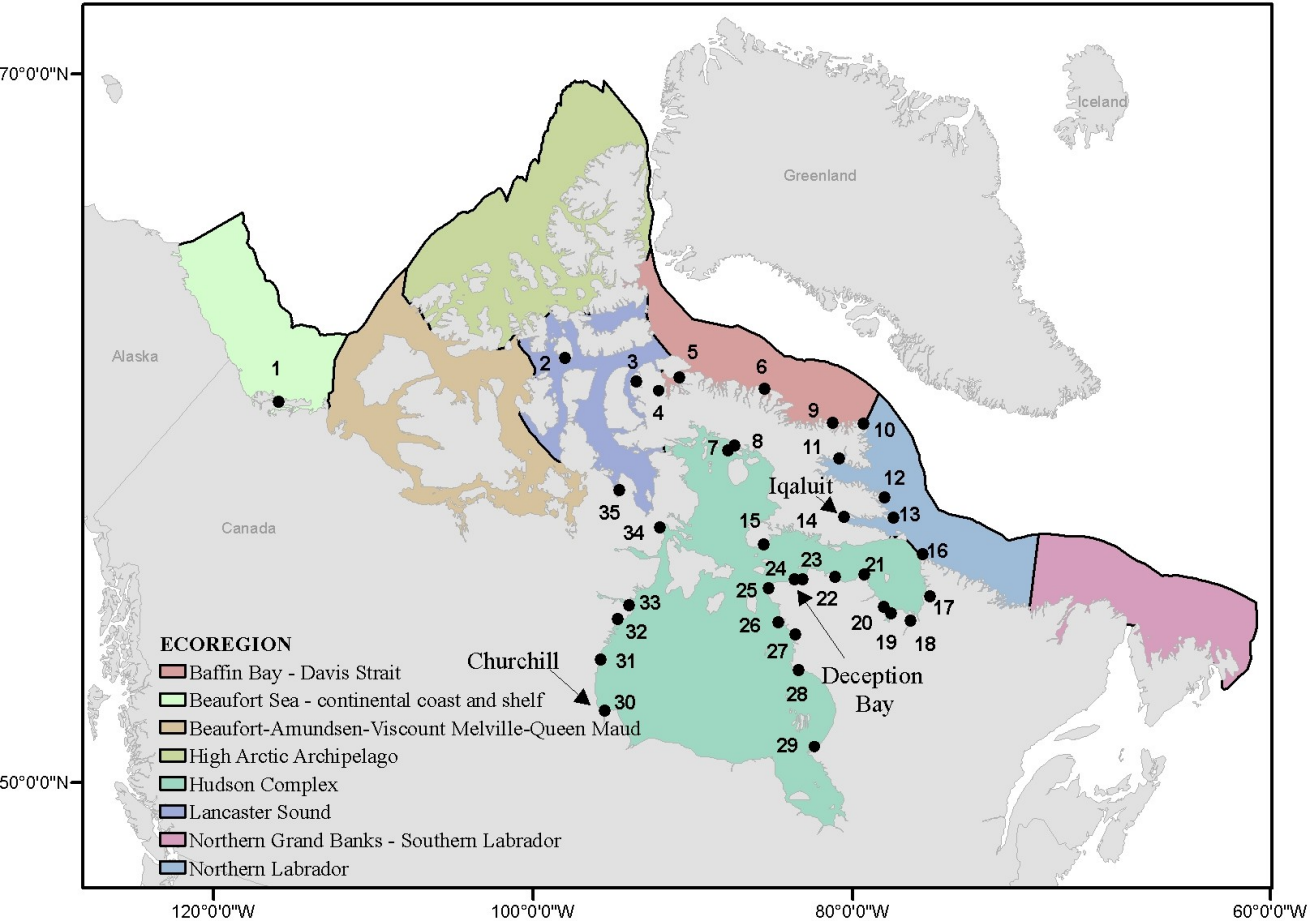
Ports considered in the present study located in the ecoregions of the Canadian Arctic. Numbers on the map correspond to port names: Tuktoyaktuk (1), Resolute Bay (2), Nanisivik (3), Milne Inlet (4), Pond Inlet (5), Clyde River (6), Hall Beach (7), Longstaff Bluff (8), Broughton Island (9), Cape Dyer (10) Pangnirtung (11), Breevoort (12), Loks Land (13), Iqaluit (14), Cape Dorset (15), Killinek (16), Kangiqsualujjuaq (17), Kujjuaq (18), Tasiujaq (19), Aupaluk (20), Quaqtaq (21), Wakeham Bay (22), Deception Bay (23), Salluit (24), Ivujivik (25), Akulivik (26), Puvirnituq (27), Inukjuak (28), Kuujjuaraapik (29), Churchill (30), Arviat (31), Rankin Inlet (32), Chesterfield (33), Repulse Bay (34), and Pelly Bay (35). Port information and ecoregions shown according to Chan et al. [60] and Spalding et al. [59].

Eight ecoregions of the Canadian Arctic, as delineated by Spalding et al. [59], were considered in this study (Fig 1). Shipping plays a key role in supporting Arctic communities, for the economy and transporting resources by domestic and international shipping. A total of 35 ports are situated in the Canadian Arctic with most in the Hudson Complex (Fig 1). Of these, Churchill, Deception Bay and Iqaluit were most actively used over the period considered in this risk assessment (2005–2014).

Churchill is located on the southwestern shore of Hudson Bay and, until its recent closure (2016), was the major seaport in the region, its main activity being the export of grain by international traffic. Churchill was previously shown to receive the highest number of vessels and volume of ballast discharge, and to be environmentally similar to a large number of connected source ports with established high risk NIS (relative to other ports in the Canadian Arctic) [60]. Shipping activity for the port of Deception Bay is related to two nickel mining sites, one of which exports concentrate to Quebec, and the other to Europe [27, 61, 62]. It is among the top 3 ports in the Canadian Arctic with respect to number of arrivals and volume of untreated ballast water released from international and coastal domestic merchant vessels. This port has high environmental similarity to a large number of its source ports, thus increasing the survival probability of NIS from linked ports [60]. Moreover, NIS have recently been found in ballast water from ships arriving to the port of Deception Bay [63, 64]. Iqaluit’s port is used for various activities: dry cargo handling (government, commercial and private use), petroleum, fisheries, tourist cruise ships, military and research vessels, Canadian Coast Guard, and small craft operators, including hunters and fishers [65]. The annual volumes of dry goods and petroleum products shipped to Iqaluit have been increasing dramatically, as have tourism and other marine activities [65]. The construction of a deep water port is scheduled to begin in 2018. Iqaluit is characterized by receiving a high level of international and coastal domestic merchant and international non-merchant vessels, and is among the top ports in the Canadian Arctic for invasion risk via hull fouling [60]. The other ports in the Canadian Arctic (Fig 1) are less active and receive mostly domestic vessels and a few international vessels with very few ballast discharge events [60]. Exceptions are ports opening with new developments that are expected to experience rapid increases in shipping over coming years [27]. Although the shipping activity in some of these ports (e.g., Milne Inlet, Nunavut, Baffinland Inc.) is expected to exceed that of current top ports in the Canadian Arctic, they are not considered within the scope of the present assessment, which relies on shipping data from the recent past.

### Species characterization

One of the first steps in this study was to identify potential NIS to use as case studies to evaluate the developed risk assessment protocol. Species were selected based on the following main factors: capacity for introduction outside of their native range, potential impacts, type and strength of ecological interactions, current distribution, and relationship with vectors (e.g., ballast water) [66]. The species included in this study (common periwinkle *Littorina littorea*; soft-shell clam *Mya arenaria;* and red king crab *Paralithodes camtschaticus*) are known to be invasive elsewhere, not present in the Canadian Arctic but present in ports that are connected to Canadian Arctic ports, and have predicted habitat suitability under present environmental conditions [32]. In addition to certain regions of the Canadian Arctic already being suitable for these three species, the predicted extent of suitable habitat will increase due to climate change [32]. The three case species are benthic invertebrates with different invasion histories and survival strategies, but all have a larval phase that is long enough so that they may be transported by ballast water (Table 1). The three species are also ecosystem engineers and thus regarded as high impact/risk species that may influence ecosystem properties and biodiversity [67].

**Table 1:** Characteristics of aquatic invasive species used in the ecological risk assessment.

Species	Native range	Non-native range	Modes of introduction	Biology / ecology	References
*Littorina littorea*	European North Atlantic.	Atlantic and Pacific coast of North America.	Rock-ballasted ships from Great Britain / Ireland in the earlies 1800s.	Herbivore. Marine and brackish. 4–7 week planktonic larval stage. Can withstand freezing. T°: -1.5 to 28°C. Salinity: 10 to 40 PSU. Intertidal.	[68–75]
*Mya arenaria*	Northwest Atlantic, from Labrador to North Carolina (uncertain limits).	Northeast Atlantic, Northeast and Northwest Pacific.	First species known to have been established in European waters. Introduced accidentally with imported seed oysters in the late 1800s to the Pacific Coast of North America.	Burrower. Bays and Estuaries. 2–3 week planktonic larval stage. T°: -2 to 28°C. Salinity: 5 to 35 PSU. Intertidal–subtidal–deeper waters.	[76–83]
*Paralithodes camtschaticus*	North Pacific, Japanese Sea and Bering Sea.	Barents Sea.	Intentional introduction in the Barents Sea to create a fishery in 1960.	Generalist predator. 2–3 month planktonic larval stage. T°: -1.7 to 11°C. Salinity: information not available. Subtidal to 300m depth.	[84–87]

### Risk characterization

**Fig 2:**
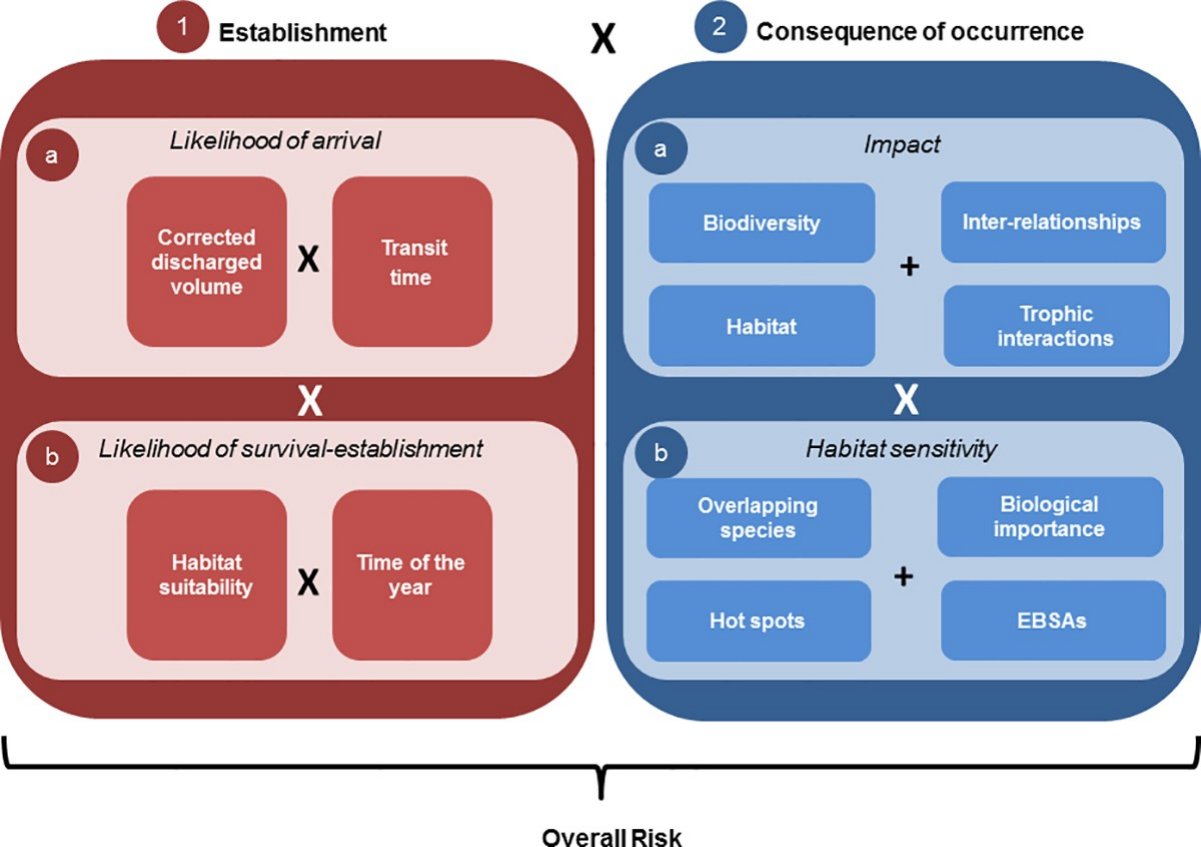
Relative risk assessment diagram. Calculation of the overall risk: 1) establishment (arrival × survival-establishment), combined with 2) consequence of occurrence (impact × habitat sensitivity).

Risk is defined as the combination of the likelihood of an event occurring and the consequences of the event if it were to occur [88]. In this study, “likelihood of an event” is defined as the likelihood of the establishment process of a non-indigenous species (a combination of introduction -arrival and release-, survival, and establishment), and “consequence” as the consequence of occurrence that a species could have if it arrives and establishes in a specific location. Overall risk is calculated as the product of establishment and consequence of occurrence per port, year, and species associated with vessel discharges (Fig 2). Methods for this relative risk assessment were adapted and modified from Hewitt et al. [89], Therriault et al. [90] and Mandrak et al. [91]. The assessment focuses on ecological effects; economic and social impacts were not considered. It must be noted that the assessment is relative, meaning that overall risk depends on the ports and species assessed.

### Establishment

The potential for successful species introduction resulting in its establishment in the region was calculated as the product of: a) likelihood of arrival (only via ballast water); and b) likelihood of survival-establishment (probability of suitable conditions and habitat being available for a given species released in the receiving environment); modified from Mandrak et al. [91] (Section *1* in Fig 2). A particular effort was made to gather detailed information on tank-specific ballast water sources, transit times, type of exchange performed and volume of ballast water discharged as outlined below.

The likelihood of arrival was restricted to shipping information for vessels that arrived at Canadian Arctic ports and reported on ballast management activities over a ten- year period (2005–2014). This information was collected from several sources: Transport Canada Ballast Reporting Database, FedNav Ballast Reporting Forms, and Casas-Monroy et al. [92]. Vessels were of both domestic (N = 75) and international (N = 178) origin and included mainly bulk carriers and merchant vessels, but also passenger ships and tugboats. The latter usually submits ballast water reports only when they carry ballast in their tanks, although reports with no ballast discharge have been submitted (P. Mudroch Transport Canada pers. comm.). Ballast discharge information was summarized by arrival port, type of BWE, pre-exchange ballast water source, and last port of call for vessel categories. When possible, data on tank-specific pre-exchange ballast water source(s) for each vessel were used for the analysis as ballast water from individual tanks can have different histories and may not originate from the last port of call. When tank-specific information was not available (N_Domestic_ = 47, N_International_ = 68), the ballast source was assumed to be the last port of call. Since this ecological risk assessment is species-specific, only ballast water sources originating from ports where the species of concern was known to be present (either in their native or non-native range) were included in the analyses described below. Both per-vessel and cumulative annual risk (based on total volumes discharged at a given port) were calculated.

Spread from the initial introduction location is normally included in the calculation of likelihood of introduction and establishment [91], given that it has an important influence as shown in other risk assessments analyses [90]. However, spread was not included in the present study since much of the required information, including high resolution data on oceanographic currents and ice-ocean modelling systems, is not available for nearshore coastal areas of the Arctic where the ports included in this assessment are located.

#### Likelihood of arrival

Relative likelihood of arrival for each vessel was estimated as the product of the volume of ballast water discharged (using a correction factor for BWE, see below) at an arrival port and the risk score for vessel transit time (section *1a* in Fig 2). Individual ballast water sources related to a given port of arrival in a given year were then combined to calculate an average likelihood of arrival per vessel, pathway (international or domestic), port, year, and species. International vessels were defined as those that operated outside of the Canadian exclusive economic zone, performed mid-ocean exchange (MOE) prior to entering Canadian waters, and are required to submit ballast water reporting information. Domestic vessels were defined as those that operated entirely within Canadian waters, and are exempt from submitting ballast water reports or performing BWE. Although these vessels are not required to perform BWE, if they do, it is typically a coastal BWE and records of ballast management activities are kept internally by the shipping companies. It has been shown that this practice may in some cases decrease BWE efficacy by increasing the abundance, and in some cases, diversity of species beyond that which was originally taken up in ballast in source ports [63, 64, 93].

A correction factor was applied to the volume discharged to account for propagule supply reduction due to ballast water management activities. To this end, ballast water discharge information was categorized according to if, and where, BWE was done. When information on the type of BWE was missing, it was assumed that international vessels had performed MOE (N = 5) and that domestic vessels did not undertake any ballast water management (N = 15). An exception was for domestic vessels known to conduct voluntary BWE as part of their regular operations [63, 64] which were assumed to have done so for all transits. In cases where a vessel was known to have discharged ballast water in a given port, but the volume was not provided, the volume discharged was assumed to be equivalent to the volume of ballast water on board (N_Domestic_ = 11, N_International_ = 38). Following categorization of BWE practices, correction factors were applied to the reported volumes of exchanged ballast water: 0.1 for ships with a saline/brackish ballast water source and 0.01 for freshwater source. These values are based on published BWE efficacy rates from total zooplankton abundance after BWE (90% for saline water and 99% for freshwater) [94, 95] and have been applied in other risk assessments [60]. Global annual mean salinity from source ports was obtained from Keller, Drake [96], and salinity categories were classified as: 0–5 ppt as freshwater, 5.1–18 ppt as brackish, and >18.1 ppt as marine [92]. When BWE was not performed, no correction factor was applied and the complete volume discharged was considered in the calculation.

The corrected discharged volume was combined with a factor for transit time, which was calculated as the difference between the date the pre-exchange ballast water was taken up at the source port and the date when the ballast water was discharged at the arrival port. Transit time was included to reflect the fact that the faster an organism reaches the destination port, the greater the chance it has of surviving the voyage and establishing a viable population in the new environment [97]. In particular, benthic taxa with a single planktonic life stage (e.g., gastropods and bivalves with planktonic larval stages) are less vulnerable to mortality in transit [98]. Details on the planktonic life stages of all case species (normal and maximal larval periods) (Table 1) were taken into consideration in ranking the transit times as low, moderate, and high (scored from 1 to 3, respectively). A low score was assigned when the transit time was longer than the maximum duration known for the larval stage of the species. Conversely, a high score was assigned when the transit time was lower than the average larval stage duration. Moderate scores were assigned to transit times that were between the average and maximum larval stage duration. In cases where information on the date of ballast water uptake was missing for transits (N_Domestic_ = 51, N_International_ = 46), an average of all other transit times was used to complete the missing information. Final values for likelihood of arrival were normalized from 0 to 1 (with 1 being the highest).

#### Likelihood of survival-establishment

Likelihood of survival and establishment was calculated as the product of habitat suitability for each species assessed and a score for the time of year when ballast water was discharged (section *1b* in Fig 2). These values were then combined to calculate an average likelihood of survival-establishment per pathway (international or domestic), port, year, and species. Habitat suitability was estimated based on the predicted suitability of regions for a given case species, resulting from species distribution modelling using MaxEnt [99, 100]. To this end, sea surface and bottom temperature, sea surface and bottom salinity, bathymetry and ice coverage were employed as environmental predictors [32]. The model predictions of habitat suitability were interpreted as likelihood of survival and establishment [101] of each species for a given region of the Arctic. To standardize results among organisms, the maximum absolute probability value generated by the model in the region of study was considered to be the highest likelihood of survival-establishment across all three species combined.

Since likelihood of survival and establishment likely vary among seasons [14, 102], the time of the year ballast water was discharged was considered. Low, moderate, and high scores were assigned when the ballast water was discharged in winter, spring/autumn, and summer according to northern hemisphere calendar seasons (ranked from 1 to 3, respectively). These ranked scores reflect the idea that most temperate species–those most likely to be transported–reproduce and recruit during the warmer seasons and would be best able to survive when waters are at their warmest [102]. This is of particular interest in the present study given that domestic and international arrivals from temperate regions represent 76% and 70% of shipping arrivals, respectively.

### Consequence of occurrence

Consequence of occurrence is defined as the potential consequence that a species may have if it is introduced in a new receiving environment. This was calculated as the product of the scores of impact and habitat sensitivity of the receiving habitat (section *2*, Fig 2).

#### Impact

Impact (section *2a*, Fig 2) is defined as a measurable change in the ecological state of an invaded ecosystem that can be attributed to NIS [103]. This includes any change in ecological or ecosystem properties. The impact that a species has had elsewhere has been shown to be a good predictor of impact in the new environment [104]. This risk component was therefore ranked based on documented information from other locations where the focal species are known to be invasive. Web of Science was used to search for documented information on each species. The name of each species was combined with “impact” and “invasion” as key words. The reported effects were divided into four categories and scored using impact rankings adapted from Hewitt et al. [89] (Table 2). These categories include: 1) changes in biodiversity, abundance and distribution, 2) changes in interspecific interactions (e.g., competition with native species for resources or space), 3) habitat (changes in the physical environment) and 4) trophic interactions (e.g., predation on native species). These four factors were each ranked from low to high (1 to 3, respectively) and summed to produce a score for impact of potential introduction.

**Table 2:** Risk component categories for consequence of occurrence.

Impact type	Low	Moderate	High
**Biodiversity, abundance and distribution**	Reduction in species richness and composition are not readily detectable.	Loss of one species. Small reduction of species richness.	Likely to cause local extinction. Loss of two or more species.
**Interspecific interactions**	No inter-relationship changes.	One kind of inter-relationship affected.	Two or more kinds of inter-relationship affected.
**Habitat**	No significant changes to habitat types.	Changes in habitat types and the habitat can be easily recovered.	Significant affected habitat area. Significant changes to habitat types.
**Trophic interactions**	No significant changes in trophic level species composition. No change in relative abundance of trophic levels (biomass).	Minor changes in trophic interactions.	Significant change in relative abundance of trophic levels and reduction of population abundances for top predator species and primary producer species.

Modified from Hewitt et al. [89].

#### Habitat sensitivity

The habitat sensitivity (section *2b*, Fig 2) criterion was used to include inherent variation in how susceptible receiving areas are to being impacted by the introduction of the novel species included in the analysis. Certain areas have been identified as biologically important in the Canadian Arctic, and this information was used to develop a proxy for habitat sensitivity. To this end, information on Ecologically and Biologically Significant Areas (EBSAs) [105] was used to determine the extent to which ports were in areas identified as possessing key ecological and biological attributes. More detailed information on certain species groups was also incorporated into the index, including: 1) overlapping species (4 or more overlapping species), 2) areas of high biological importance (highly productivity areas due to particular conditions), and 3) hot spots and areas of special interest (areas of high diversity and/or high biomass) following the approach outlined in Stewart et al. [106] and Goldsmit et al. [107]. Although this latter data set is biased toward harvested species, it was reasoned to be the best available proxy for areas of particular biological importance that could be more sensitive to the arrival of NIS. To rank ports for habitat sensitivity, each was evaluated to determine the degree to which it overlapped with the spatial distribution of these four variables (EBSA, overlapping species, high biological importance and hot spots). Ports that overlapped with one, two to three, or four sensitivity variables were considered to have low, moderate, and high habitat sensitivity, respectively, and were assigned scores from 1 to 3, respectively.

### Overall risk

All components described above were combined to evaluate overall risk as shown in Fig 2. Prior to determining overall risk, establishment and the consequence of occurrence were normalized from 0 to 1, using the minimum and maximum values across all three species combined, to standardize results among organisms and ports.

The normalized values for these two risk components were combined in a risk matrix depicted using a gradient approach to indicate overall risk [91]. Risk matrices were constructed for each species by arrival port and year, for both domestic and international transits per vessel, and per cumulative annual discharges. The use of this gradient approach enables illustration of the continuous nature of overall risk both spatially (ports) and temporally (years) along gradients of likelihood and consequences for each species [91].

### Uncertainty

The strength of a risk assessment is dependent on the uncertainty associated with the data [91] and must be explicitly considered for each step of the risk assessment based on the extent of available information and gaps. Three types of uncertainty exist: stochastic, imperfect knowledge, and human error. In this study, the greatest uncertainty affecting the assessment is imperfect knowledge, namely, lack of knowledge. The quality and quantity of data available to assess establishment process and magnitude of consequences needs to be incorporated in uncertainty [101]. Uncertainty was considered in each step of the risk assessment according to the availability and type of information used, as modified from Therriault et al. [90]. As suggested in Mandrak et al. [91], it was included with the risk ranking by describing the amount of information available, but it was not incorporated into the scores. Uncertainty was considered high when limited scientific information was available and low when the analysis was based on substantial scientific information. It was also considered low when quantitative methods, such as the habitat suitability modelling used to calculate the likelihood of survival/establishment, were included in the risk assessment. Uncertainty was considered moderate when there was a moderate level of peer-reviewed information and expert opinion available. Overall uncertainty was considered to be equivalent to the highest uncertainty associated with any variable used in the analysis [91].

## Results

### General shipping results

Among vessels that conducted ballast management activities from 2005 to 2014, Deception Bay received the highest average number of domestic arrivals annually (7.5), followed by Churchill (3.6) and Iqaluit (2.5) (Table 3). For international vessels, that conducted ballast management, Churchill received the highest average annual number of arrivals (16.1), followed by Pond Inlet (3.5) and Iqaluit (2.5) (Table 3). Overall, 93.3% of domestic ships and 70.8% of international ships for which ballast reporting information was available, discharged their ballast at Canadian Arctic ports (for a complete list of results see Table 3, and refer to Fig 1 for port geographic locations). Of these, pre-exchange ballast water sources differed from the last visited ports for 11.1% and 31.1% of domestic and international arrivals, respectively.

**Table 3:** Domestic and international transits with information on the corrected ballast water discharged.

Domestic arrival ports	Year	N° of arrivals per year	N° of vessels that discharged ballast water	Mean (±SD) corrected ballast water discharged/vessel (MT)	Total corrected discharge (year/port) (MT)
**Aupaluk**^**20**^	2005	1	1	3971 (0)	3971
	Port mean/year	1	1	**3971 (0)**	Total discharged: 3971
**Broughton Island**^**9**^	2005	1	1	3971 (0)	3971
	Port mean/year	1	1	**3971 (0)**	Total discharged: 3971
**Chesterfield**^**33**^	2007	1	0	0	0
	Port mean/year	1	0	0	Total discharged: 0
**Churchill**^**30**^	2005	4	4	11257.3 (7844.7)	45029.4
	2006	7	7	1486.6 (933.6)	10406
	2007	1	1	1607.4 (0)	1607.4
	2013	3	3	10736.8 (3534.7)	32210.4
	2014	3	3	4480.5 (5119.6)	13441.6
	Port mean/year	**3.6**	3.6	**5705.3 (6295.9)**	Total discharged: **102694.77**
**Clyde River**^**6**^	2010	1	0	0	0
	Port mean/year	1	0	0	Total discharged: 0
**Deception Bay**^**23**^	2005	6	6	10253 (0)	61518
	2006	6	6	10253 (0)	61518
	2007	7	4	7542.1 (4161.9)	30168.3
	2008	10	10	9273.6 (2042.8)	92736
	2013	10	10	8263.9 (2010)	82639.1
	2014	8	8	9153.6 (1215.6)	73229
	Port mean/year	**7.5**	7.3	**8549.1 (3033.2)**	Total discharged: 401808.4
**Inukjuak**^**28**^	2005	1	1	3384 (0)	3384
	Port mean/year	1	1	3384 (0)	Total discharged: 3384
**Iqaluit**^**14**^	2005	3	3	3775.3 (276.7)	11326
	2006	2	2	3365.5 (605.5)	6731
	Port mean/year	**2.5**	2.5	3611.4 (482.6)	Total discharged: **18057**
**Kuujjuaraapik**^**29**^	2005	1	1	6.9 (0)	6.9
	2007	1	1	3384 (0)	3384
	Port mean/year	1	1	1695.5 (1688.6)	Total discharged: 3390.9
**International arrival ports**					
**Broughton Island**^**9**^	2010	1	0	0	0
	Port mean/year	1	0	0	Total discharged: 0
**Cape Dyer**^**10**^	2007	1	1	397.1 (0)	397.1
	Port mean/year	1	1	**397.1 (0)**	Total discharged: **397.1**
**Chesterfield**^**33**^	2010	1	0	0	0
	Port mean/year	1	0	0	Total discharged: 0
**Churchill**^**30**^	2005	12	12	632.2 (480.3)	7586.7
	2006	12	10	1343 (1023.5)	13430.4
	2007	21	18	861.9 (471.9)	15514.2
	2008	18	17	875.3 (630.1)	14879.8
	2009	17	17	548.7 (382.3)	13718
	2010	22	20	1116.3 (498)	22325.1
	2013	14	14	1103.7 (780.9)	15452.2
	2014	13	13	979.7 (642.3)	12736.6
	Port mean/year	**16.1**	15.1	**955.7 (630.6)**	Total discharged: **115642.9**
**Clyde River**^**6**^	2010	1	0	0	0
	Port mean/year	1	0	0	Total discharged: 0
**Deception Bay**^**23**^	2007	2	1	1494.6 (0)	1494.6
	2008	1	0	0	0
	2009	2	1	0.54 (0.54)	0.5
	2010	1	0	0	0
	2011	1	1	23.4 (0)	23.4
	2013	1	1	45.1	45.1
	Port mean/year	1.3	0.7	**390.9 (637.4)**	Total discharged: **1563.7**
**Iqaluit**^**14**^	2007	4	0	0	0
	2008	3	0	0	0
	2009	1	0	0	0
	2010	2	0	0	0
	Port mean/year	**2.5**	0	0	Total discharged: 0
**Kuujjuak**^**18**^	2010	1	0	0	0
	Port mean	1	0	0	Total discharged: 0
**Pangnirtung**^**11**^	2008	1	0	0	0
	2009	2	0	0	0
	Port mean/year	1.5	0	0	Total discharged: 0
**Pond Inlet**^**5**^	2006	2	0	0	0
	2008	4	0	0	0
	2009	3	0	0	0
	2010	5	0	0	0
	Port mean/year	**3.5**	0	0	Total discharged: 0
**Rankin Inlet**^**32**^	2009	1	0	0	0
	Port mean/year	1	0	0	Total discharged: 0
**Resolute Bay**^**2**^	2008	1	0	0	0
	Port mean/year	1	0	0	Total discharged: 0
**Tuktoyaktuk**^**1**^	2008	4	0	0	0
	2009	2	0	0	0
	2010	1	0	0	0
	Port mean/year	2.3	0	0	Total discharged: 0

Ports with highest average number of arrivals and vessel-specific quantities of corrected ballast water discharged and total discharge per port are highlighted in bold. The information included in the table reflects years and arrivals where complete data were available. The reference number of each port in Fig 1 is shown as superscript beside the name. All information included in this table is according to the data available during the years 2005–2014 from the following data sources: Transport Canada, FedNav Ballast Reporting Forms, and Casas-Monroy et al. [92].

Mean corrected ballast water discharge for domestic arrivals was highest for Deception Bay, Churchill, Aupaluk and Broughton Island, while total discharges were higher for Deception Bay, Churchill, and Iqaluit (Table 3). For international arrivals, the mean and total corrected ballast water was highest for Churchill, followed by Cape Dyer and Deception Bay (Table 3).

For domestic vessels ports that had the highest number of arrivals also had the highest total corrected discharge per port (Table 3). This was not the case for international arrivals, since many vessels arrived but did not discharge ballast in the Arctic. Thus, Deception Bay had the second highest total discharge of ballast water from international vessels although it only had a mean of 1.3 arrivals per year (Table 3).

### Establishment

#### Likelihood of arrival

**Fig 3:**
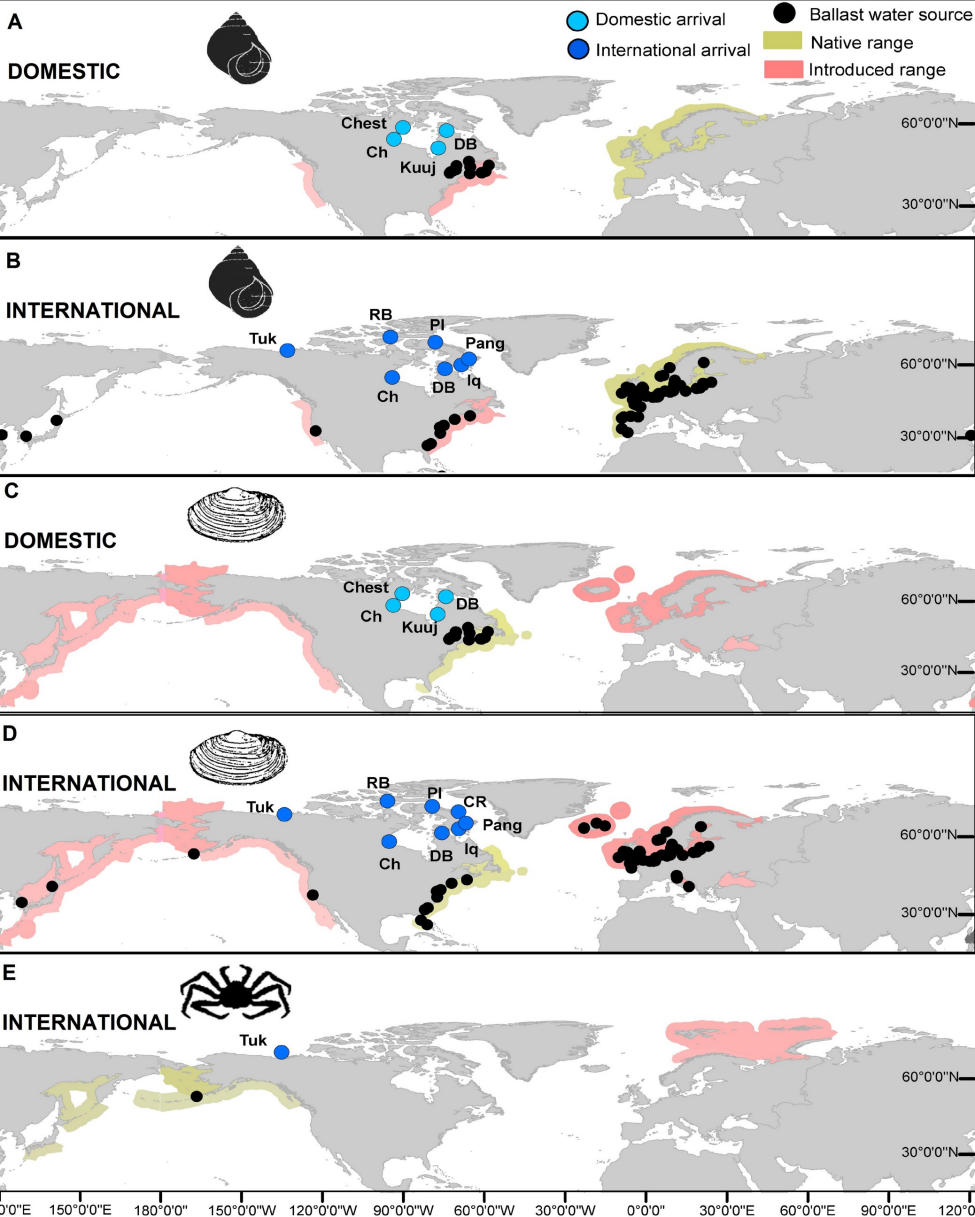
Ports of arrival for the Canadian Arctic for ships coming from regions where the assessed species are present. A) Domestic arrivals *Littorina littorea*, B) international arrivals *Littorina littorea*, C) domestic arrivals *Mya arenaria*, D) international arrivals *Mya arenaria*, E) international arrivals *Paralithodes camtschaticus*. Port names are shown as following: Churchill (Ch), Chesterfield Inlet (Chest), Clyde River (CR), Deception Bay (DB), Iqaluit (Iq), Kuujjuaraapik (Kuuj), Pangnirtung (Pang), Pond Inlet (PI), Resolute Bay (RB), Tuktoyaktuk (Tuk).

Four ports received vessels with domestic ballast water originating from regions where both the periwinkle *L*. *littorea* and the soft shell clam *M*. *arenaria* are present (Fig 3A and 3C). Among these, Deception Bay (years 2005, 2006, 2008, 2013 and 2014) and Churchill (year 2005) received the highest corrected volumes of discharged ballast water per vessel for both species (S1 and S2 Tables). Ballast water age (considered here as transit time) varied from 5 to 37 days in domestic vessels, resulting in a high score for *L*. *littorea* with a maximum known larvae stage of 7 weeks (49 days), and a moderate score for *M*. *arenaria* with a maximum known larvae stage of 3 weeks (21 days). Seven and eight ports received vessels with ballast water originating from international regions where *L*. *littorea* and *M*. *arenaria* are present, respectively (Fig 3B and 3D). Among these, Churchill received the highest volumes of species-specific corrected discharged ballast water per vessel. However, since most vessels performed MOE, the corrected ballast water volumes that were discharged were lower for all years (2005 to 2014) when compared to domestic discharges (S3 and S4 Tables). Other ports received low volumes or no ballast water from international sources where these species are known to occur (S3 and S4 Tables). Ballast water age for international vessels varied from 5 to 135 days, such that all three types of scores (high, moderate and low) for transit time were recorded for both species. For red king crab *P*. *camtschaticus*, only the port of Tuktoyaktuk is connected to an international port where the species is present (Fig 3E, S5 Table) and ballast from that port was not discharged there. Uncertainty in this section was considered to be moderate due to the assumptions that were made to complete the database of shipping arrivals and ballast water discharges.

#### Likelihood of survival-establishment

**Fig 4:**
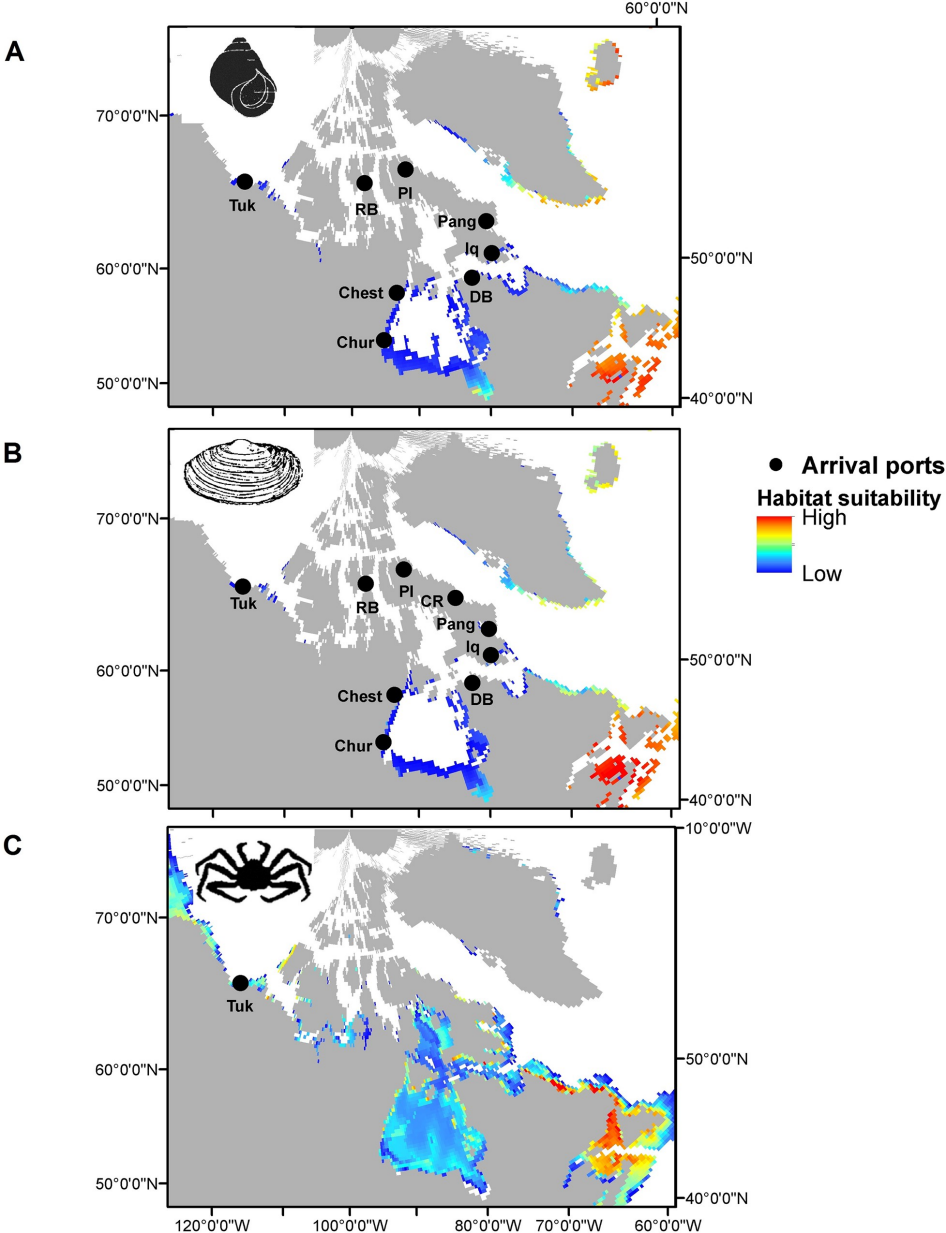
Likelihood of survival-establishment based on habitat suitability under current environmental conditions. A) *Littorina littorea*, B) *Mya arenaria*, and C) *Paralithodes camtschaticus*. All colored areas are to some extent suitable for the species (modified from Goldsmit et al. [32]).

The likelihood of species survival-establishment based on species distribution modelling under current environmental conditions is shown in Fig 4. For *L*. *littorea* and *M*. *arenaria*, even though the probabilities are low, there are many coastal areas where the habitat is suitable, although only a few ports, including Resolute Bay and Pond Inlet, are situated in such areas. In contrast, habitat suitability for *P*. *camtschaticus* is generally much greater and much more wide-spread throughout the Canadian Arctic. However, there is currently only one potential port of arrival for this species. Uncertainty in this section was considered to be low given that it is based on substantial information and proven quantitative methodology.

### Consequence of occurrence

#### Impact

Evidence for impacts of the species assessed in this study is given in detail in S6 Table. All three species have known effects in other environments in all four impact categories. Potential impact scores varied from moderate to high, depending on the species and category, with *P*. *camtschaticus* having the highest overall combined impact score. Uncertainty in this section was considered to be low given the substantial available scientific information.

#### Habitat sensitivity

The ports with highest sensitivity were Deception Bay, Pangnirtung, and Resolute Bay (S1 Fig). All other ports (Churchill, Iqaluit, Chesterfield, Clyde River, Pond Inlet and Tuktyaktuk) that received domestic or international vessels had moderate sensitivity. None of the ports considered in this section of the study were characterized as having low habitat sensitivity. Uncertainty for habitat sensitivity was considered to be low given that the information used in this section was based on substantial published scientific information for the study region.

### Overall risk

**Fig 5:**
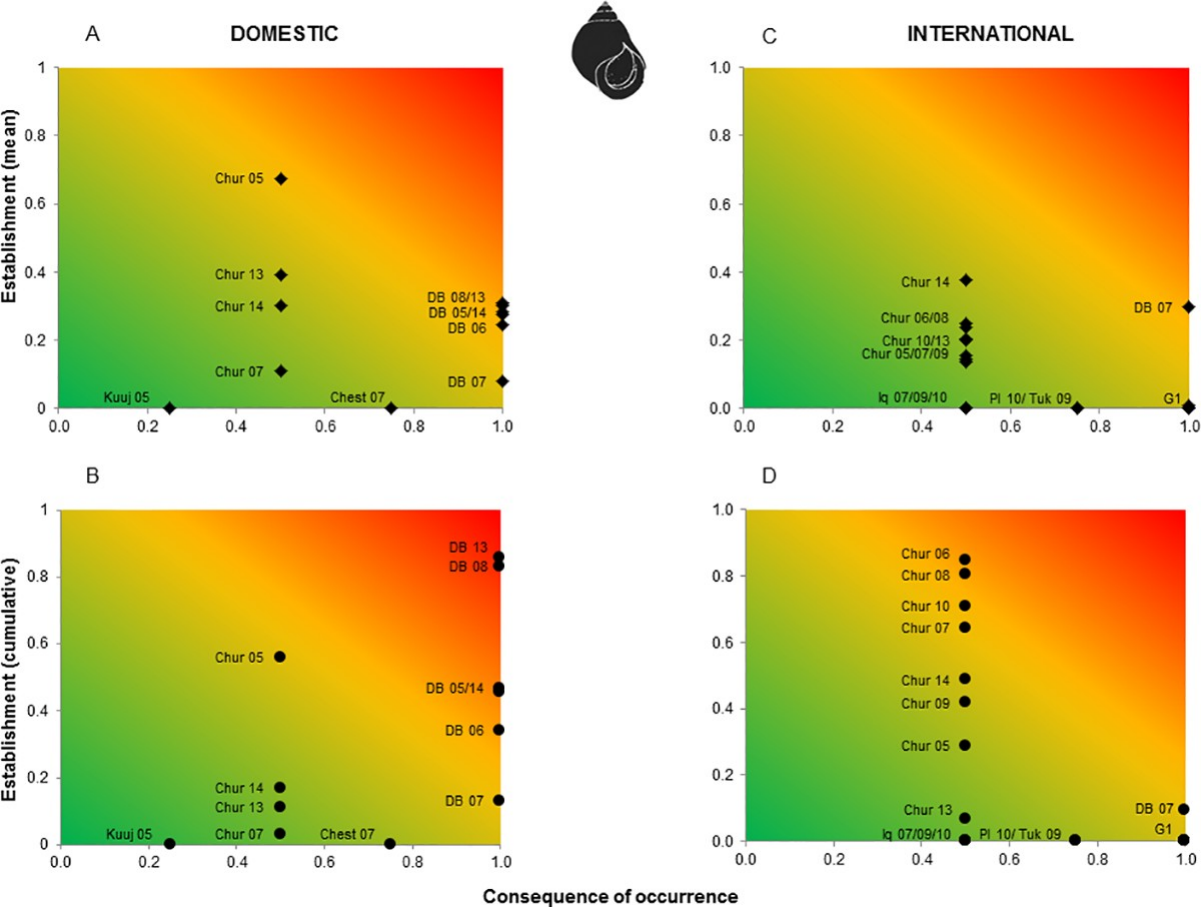
Overall risk matrices (risk as a function of establishment process and consequence of occurrence) depicted as a gradient showing the differences between ports and years for *Littorina littorea*. A) Risk per vessel per port due to domestic pathways, B) annual cumulative risk per port due to domestic pathways, C) risk per vessel per port due to international pathways, and D) annual cumulative risk per port due to international pathways. Colors represent overall risk associated with vessel discharges: Low (green), moderate (yellow) and high (red). Port names are shown as following: Chesterfield (Chest), Churchill (Chur), Deception Bay (DB), Iqaluit (Iq), Kuujjuaraapik (Kuuj), Pond Inlet (PI), Tuktoyaktuk (Tuk). G1 is a group of port/years having low risk and values being all close to each other. G1 includes: Deception Bay 2008, 2010 and 2011, Pangnirtung 2009, and Resolute Bay 2008.

**Fig 6:**
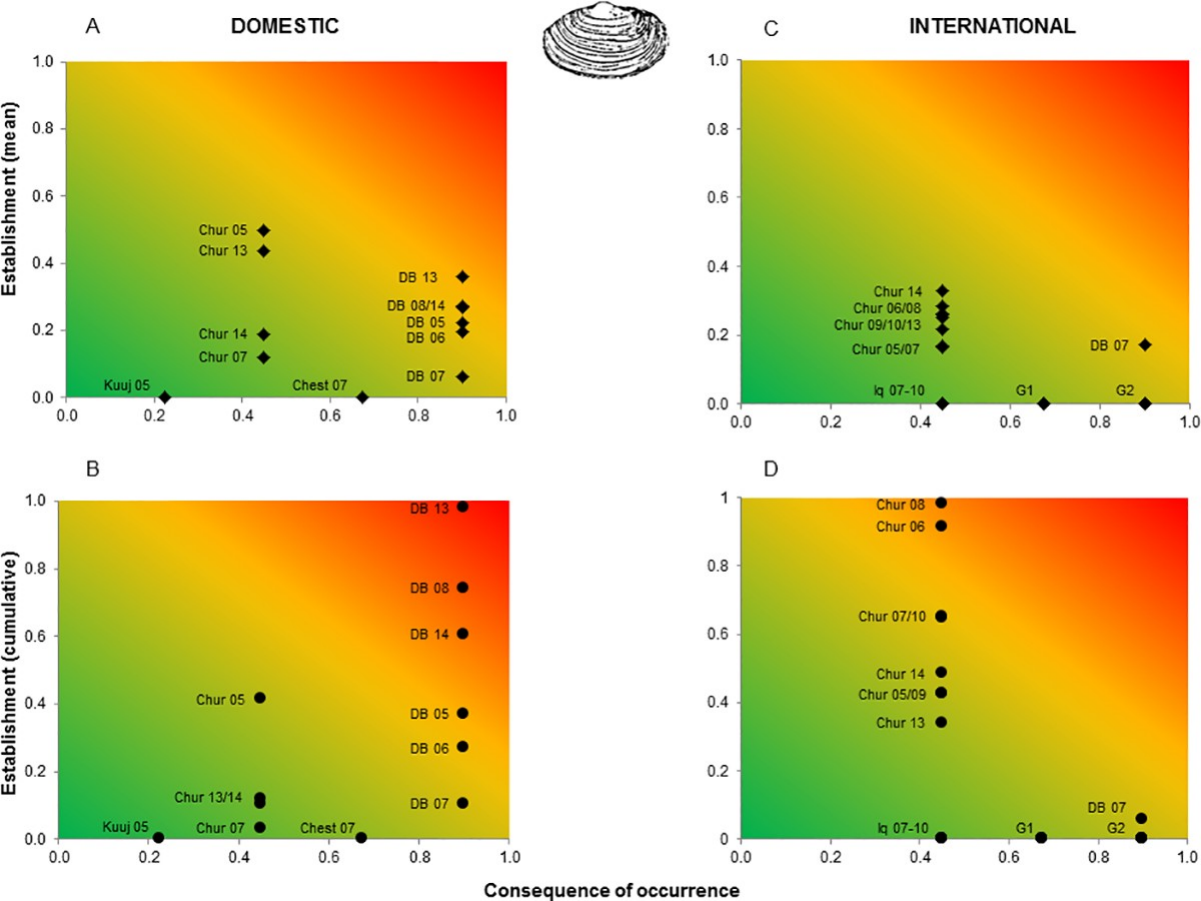
Overall risk matrices (risk as a function of establishment process and consequence of occurrence) depicted as a gradient showing the differences between ports and years for *Mya arenaria*. A) Risk per vessel per port due to domestic pathways, B) annual cumulative risk per port due to domestic pathways, C) risk per vessel per port due to international pathways, and D) annual cumulative risk per port due to international pathways. Colors represent overall risk associated with vessel discharges: Low (green), moderate (yellow) and high (red). Port names are shown as following: Chesterfield (Chest), Churchill (Chur), Deception Bay (DB), Iqaluit (Iq), Kuujjuaraapik (Kuuj). G2 and G3 are groups of port/years having low risk and values being all close to each other. G2 includes: Clyde River 2010, Tuktoyaktuk 2008 and 2009. G3 includes: Deception Bay 2008, 2010 and 2011, Pangnirtung 2009, and Resolute Bay 2008.

Overall risk varied greatly among ports, years, species and pathways (Figs 5 and 6). Neither mean per vessel or cumulative annual discharges showed increasing trends. However, domestic discharge events tended to pose greater relative overall risk than did international discharge events both per vessel and on an annual cumulative basis. Cumulative annual risk was generally higher than mean risk per vessel with the exception of Churchill 2013 and 2014 for domestic vessels and Deception Bay 2007 for international vessels. In particular, vessels that discharged in the port of Deception Bay posed the highest overall cumulative annual risk for domestic arrivals, followed by those that discharged ballast water in the port of Churchill. For international arrivals, the highest relative annual cumulative risk was for vessels discharging in the port of Churchill. On a relative scale, mean risk per vessel discharge in a given port was at times lower than was the corresponding cumulative annual value. This may reflect that a small number of vessels with much ballast may discharge a larger total quantity than a greater number of ships with little ballast (e.g. international vessels arriving at Churchill 2006 compared to Churchill 2014 for *L*. *littorea* and *M*. *arenaria*, Figs 5 and 6).

The patterns of overall risk associated with discharges from domestic vessels were similar for *L*. *littorea* and *M*. *arenaria*. For *L*. *littorea*, risk per vessel was variable for Churchill, with moderate to high overall risk in 2005, and low to moderate risk in all other years. In contrast, risk per vessel for Deception Bay was relatively stable through time, at moderate to high from 2005 to 2014 (Fig 5A). Most ports receiving international vessels from regions where the target species is present had low relative overall risk per vessel, with the exception of Deception Bay in 2007, where relative overall risk was low to moderate (Fig 5C). When considered on an annual cumulative basis Deception Bay had a higher overall risk for domestic vessels over most years, while Churchill tended to have a higher overall risk for international vessels in most years with the exceptions of 2005 and 2013 (Fig 5B and 5D). For *M*. *arenaria*, domestic vessels arriving at Churchill varied between years, ranging from low to moderate risk per vessel. Relative risk to Deception Bay generally increased from low (2007) to moderate (2005, 2008–2014) (Fig 6A). For international arrivals, most ports showed a low relative overall risk, with the exception of Deception Bay in 2007, which was moderate (Fig 6C). Deception Bay had the highest overall risk for domestic vessels on an annual cumulative basis ranging from moderate to high, while Churchill tended to have the highest overall risk for international vessels across most years (Fig 6B and 6D). Although some other ports could be highly impacted, the probability of establishment of *M*. *arenaria* is generally low for international vessels (with the exception of Churchill), resulting in decreased overall risk (Fig 6D). Only one port in the Canadian Arctic was connected to a region where *P*. *camtschaticus* is present and it only received one international ship, on a single occasion, which did not discharge ballast. Thus, no risk matrix is shown for this species.

The uncertainty associated with establishment process was moderate (combination of moderate uncertainty for likelihood of arrival and low uncertainty for likelihood of survival/establishment), and low for consequence of occurrence (combination of low uncertainty for impact and low uncertainty for habitat sensitivity). Hence, the overall uncertainty for overall risk was moderate.

## Discussion

This relative risk assessment provides information on the potential risk of introduction, establishment, and impact for three species that are not, to our knowledge, currently present in the Canadian Arctic, but for which there is likely suitable habitat for their survival and establishment in the region. The methodology used in the present study is unique in that it considers ballast water sources and the distribution of NIS (i.e., the potential availability of NIS propagules in ballast water sources). Moreover, it evaluates the relative risk of the combination of each port, year, and species considered, thus allowing for a more realistic, comprehensive ecological risk assessment at the species-level. The results show that ports in the Canadian Arctic have likely been exposed to propagules of NIS that are established in connected ports or BWE locations, especially via domestic vessels. Although the current probabilities of establishment for the species considered in this study are generally low, it is important to note that the consequence of their establishment ranges from moderate to high for most ports considered in the study. Thus, if vessel-specific ballast water discharges increase in the future, so too will relative overall risk. This is a plausible scenario given that shipping activity in the Canadian Arctic is expected to increase in the future due to the opening of seasonal trading routes through the North West Passage and increasing resource exploitation in the region [25, 27, 28]. The level of risk could be also influenced by projected increases in the habitat suitability for these species in the region as a result of global climate change [32].

Mean and cumulative ballast water discharges were temporally and spatially variable such that potential for introduction was not uniform among Canadian Arctic ports, consistent with findings of Chan et al. [108]. On the other hand, potential impacts varied more by species and location. Thus, overall risk of vessel discharges may fluctuate according to location, time, and species when all factors are considered. In general, the Hudson Bay Complex can currently be considered to be at higher relative risk compared to the other regions in the Canadian Arctic. This is due to the region receiving a greater proportion of vessels coming from regions where the species of concern are present, the type of exchange performed, and because most vessels’ destination ports are in this area. Moreover, ports in the region have higher environmental similarity with their connected ports relative to ports in other Canadian Arctic regions [60] and thus could provide suitable habitat to potential NIS [32]. In particular, vessel discharges in Deception Bay were found to pose greater overall risk across most years and species, relative to other ports receiving a higher number of vessel discharges, such as Churchill, because of the unique combination of ballast history/discharges and consequences of occurrence.

Management actions vary by vessel origin. International vessels are required to perform MOE prior to entering Canadian waters. In contrast, as outlined in the Ballast Water Control and Management Regulations (SOR/2011-237), domestic vessels operating within Canadian waters are exempt from BWE requirements, although some do so on a voluntary basis. Depending on the source port, domestic vessels that do not conduct BWE may transport large volumes of ballast from other marine regions of Canada that may include some of the NIS considered in this study. Discharge of un-exchanged ballast water can thus represent an increase in the probability of establishment of these NIS species due to a higher actual likelihood of arrival [109]. However, domestic vessels originating from freshwater ports and undertaking voluntary ballast water exchange in brackish/saline coastal waters may inadvertently increase the probability of propagules of the marine species considered in the present study of arriving and which would not otherwise have been present in the original freshwater ballast [63, 64]. Hence, risk is expected to vary among ports as a function of source, discharge, and treatment of ballast water, in agreement with Verling et al. [110] and Cordell et al. [17]. However, successful invasion may require multiple introductions [111] such that frequency of propagule supply [112], together with the timing, volume, and location of ballast water release may play a fundamental role. This is of importance as this study shows that some ports receive frequent but low volumes of ballast water. The repetitive discharge of ballast water increases risk as reflected by the annual cumulative ballast water discharge. These factors may all be more important to introduction success than is the number of organisms contained in the released water [113]. Notwithstanding this, the number of propagules released in a given event may also be important, since the greater the number of individuals released, the more likely some will survive stochastic events [97]. In any case, the combination of environmental conditions must be appropriate for released individuals to establish [114].

Ballast water release and hull fouling are thought to be the most important vectors for the introduction of aquatic organisms [13, 115]. Therefore, accurate ship history is of great importance to assess risk of ballast discharge. Importantly, distinguishing between the last port of call and the ballast water source, as was done in this risk assessment, should logically increase the accuracy of assessing the risk of any given discharge event and, when available, this information should be used. This is particularly important when assessing the risk of a given species for which distributional data is available to evaluate environmental similarity donor and receiving ports [66]. If ballast origin is incorrectly attributed, risk assessments may be misleading. To our knowledge, no other pathway risk assessment study has considered ballast water source differently from the source port. Another important component of ship history is transit time (time since ballast uptake until it is discharged) which impacts biological communities in ballast water [116]. Natural mortality in ballast water tanks has been observed [14] and, all else being equal, proximity of donor region and ballast water age will affect propagule conditions [97], such that propagules that spend less time in ballast will be more able to survive transit and establish. Despite propagule mortality due to ballast water treatment, degrading conditions, and natural senescence, some individuals may continue to survive transits, as shown by sampling organisms in ballast water upon arrival in receiving ports [97]. In particular, benthic invertebrates that spend only part of their lives as plankton (e.g., gastropods and bivalves) appear to be less vulnerable to mortality *en route* [98]. Thus, although it is not possible to predict when arrivals may occur, a precautionary approach is recommended given the possibility of propagules being discharged in recipient ports [66]. This study has also shown that some ports that receive international vessels do not receive any ballast water. Such vessels most likely transport cargo to communities and appear to mainly originate from other cold/Arctic regions. In fact, this type of behavior could mean that ballast is being taken up in Canadian Arctic ports and transported elsewhere, thus becoming a potential source of NIS elsewhere, as highlighted in Goldsmit et al. [38].

Predicting the establishment of a species in an environment needs to be carefully evaluated by considering life stages, seasonal variations, and abiotic tolerances [54, 66]. The present risk assessment took all these factors into consideration in the overall risk calculation by including transit time relative to the length of planktonic stage for each species and the season when the ballast water was discharged. These factors, when combined with the use of predicted habitat suitability, should improve risk assessment precision, allowing analyses to be done at a species-level. The present study assessed the overall risk of two mollusks (*Littorina littorea* and *Mya arenaria*) and one crustacean (*Paralithodes camtschaticus*). A common characteristic of these three species is that they all include a long-lived feeding planktonic larval period. Larval ecology (i.e., planktotrophic larvae versus short-lived non-feeding ones or lecitotrophic larvae) may influence how dispersal rates vary for organisms with different reproductive strategies [117]. The risk of introduction may be affected by the fact that some species can delay metamorphosis in the absence of suitable substrate for settlement, thus extending their planktonic larval phase from weeks to months [118]. This may increase risk of introduction as such larvae may survive extended periods by feeding in the water column. In addition, MOE is not always effective for certain species, including *L*. *littorea* and *M*. *arenaria* [116]. For these two species in the current risk assessment, the overall risk was higher for discharges from domestic rather than international arrivals. Given that both are presently distributed in regions where the coastal exchange of ballast water of domestic vessels was performed, the management action in this case is likely increasing risk. Although ballast water exchange logically reduces the likelihood of arrival of new species, in some cases, the efficacy of ballast water exchange as a mitigation strategy is questionable [4, 119–122]. In contrast, the likelihood of *P*. *camtschaticus* arrival by domestic transits was null and was low for international transits, but the likelihood of survival-establishment and consequence of occurrence of this species is considered to be very high, highlighting the need for extreme caution if the likelihood of arrival increases in the future. Interestingly, trans-Arctic exchange of species is expected in the future [123] and environmental niche modelling suggests that most Canadian Arctic regions are suitable for this species [32]. Given these predictions, the risk of introduction could be then increased by marine transportation or from natural dispersion via currents or migration. There is evidence that some shallow water organisms have been able to extend their ranges from the Bering Sea to the Atlantic as a results of warmer Arctic conditions [124].

In general, known consequences of a species in one location are good predictors of consequences in new non-native ranges and this information is commonly used in risk assessments [104, 125]. The most widely documented consequences include declines in native populations, altered nutrient cycling, food web alterations with changes in competitor and consumer pressure, and physical habitat changes [126–128]. There is no way to precisely predict the impact that a given NIS will cause in a new environment unless it becomes established [66, 129]. The consequence of occurrence assessed in the present study included the combination of the known consequences of each species when it had established elsewhere and the sensitivity of receiving habitats. Impacts are species-specific, while habitat sensitivity is port-specific. The latter is essential to include in these types of assessment as it is reasoned that the severity of consequences will also be a function of receiving habitat characteristics [130]. In the present study, most ports showed moderate to high potential consequence of occurrence. If impacts and habitat sensitivity remain constant, the overall risk will increase as the probability of establishment process increases, varying with ballast water source and the species assessed. This demonstrates the importance of preventing the introduction of new species and highlights the need for good management actions and preventive measures for ballast water management in the region.

In this study, a particular effort was made to gather detailed information on the number of arrivals, tank-specific ballast water sources, transit times, type of exchange performed and volume of ballast water discharged. However, it should be noted that, under the new IMO regulations which came into force in September 2017 [131], ships arriving from outside of Canada will be required to treat ballast with an approved treatment system (e.g., filtration, with chlorination and/or UV irradiation). While this is not yet mandatory for all vessels, may not be effective for all species [92], and efficacy under different environmental conditions, particularly in colder waters, is not well understood [132]. Under future conditions when more vessels are conducting treatment and when efficacy is better understood, the methodology proposed in the current assessment should be revised together with treatment-specific correction factors used to weight the known volume of ballast water discharged and/or treated.

This assessment only considered risks associated with ballast water, however other vectors directly related to shipping such as biofouling and ballast sediments, are also potential sources of NIS. Thus, the actual overall risk for a species may be even greater if it is associated with hull fouling [34, 133], hull refuges, including sea chests [134], or with ballast sediments, which have been shown to include viable resting stages of many species with the potential of being released during de-ballasting in the receiving port due to resuspension [135–137]. However the complete history of these kinds of vectors should be captured (not only last port of call) and their importance will depend on the species being assessed and their life histories. While an important consideration, adequately characterizing history for these types of vectors requires going back several steps (multiple ports of call) and was therefore beyond the scope of this study.

The ecological risk assessment protocol developed in the present study allowed for assessment of ports through time and enabled comparison between species, shipping pathways, and volume of ballast water discharge. Using this detailed methodology can help identify the relative risks of key entry points and pathways for early detection monitoring [101]. Currently, many countries are developing blacklists (i.e. lists of NIS with presumed invasive potential in the area of interest) [138]. These lists are developed with the aim of preventing introductions of new harmful species and regulating the spread of species that are already present in a given region [139]. Recently, “grey” watch lists, which contain species of potential risk [140], have also been developed. The present ecological risk assessment can provide a starting point to build a grey watch list of NIS for the Canadian Arctic. This ecological risk assessment is the first to incorporate detailed shipping vector information at a species specific level, allowing for comparison of risk across pathways and locations over time. Although, only three species were assessed in this particular study, the proposed methodology may be used for any species of interest and provides an ideal tool for comprehensively assessing the relative risk of potential NIS arriving in areas that have not yet been invaded. Such information can help guide prevention and management efforts in frontier regions where knowledge is lacking, such as the Canadian Arctic.

## Supporting information

S1 Fig Ports showing locations and habitat sensitivity according to the overlap of sensitivity variables. (PDF)

S1 Table Complete information on ballast water discharged at each Canadian Arctic port through domestic vessels with ballast water from regions where *Littorina littorea* is present. Volumes are given in metric tons (MT). Correction factor for ballast water exchange: 1 (no exchange), 0.1 (mid ocean exchange (MOE), considered for ships with a saline/brackish ballast water source), 0.01 (MOE for ships with freshwater ballast water source).
(DOCX)

S2 Table Complete information on ballast water discharged at each Canadian Arctic port through domestic vessels with ballast water from regions where *Mya arenaria* is present. Volumes are given in metric tons (MT). Correction factor for ballast water exchange: 1 (no exchange), 0.1 (mid ocean exchange (MOE), considered for ships with a saline/brackish ballast water source), 0.01 (MOE for ships with freshwater ballast water source).
(DOCX)

S3 Table Complete information on ballast water discharged at each Canadian Arctic port through international vessels with ballast water from regions where *Littorina littorea* is present. Volumes are given in metric tons (MT). Correction factor for ballast water exchange: 1 (no exchange), 0.1 (mid ocean exchange (MOE), considered for ships with a saline/brackish ballast water source), 0.01 (MOE for ships with freshwater ballast water source).
(DOCX)

S4 Table Complete information on ballast water discharged at each Canadian Arctic port through international vessels with ballast water from regions where *Mya arenaria* is present. Volumes are given in metric tons (MT). Correction factor for ballast water exchange: 1 (no exchange), 0.1 (mid ocean exchange (MOE), considered for ships with a saline/brackish ballast water source), 0.01 (MOE for ships with freshwater ballast water source).
(DOCX)

S5 Table Complete information on ballast water discharged at each Canadian Arctic port through international vessels with ballast water from regions where *Paralithodes camtschaticus* is present. Volumes are given in metric tons (MT). Correction factor for ballast water exchange: 1 (no exchange), 0.1 (mid ocean exchange (MOE), considered for ships with a saline/brackish ballast water source), 0.01 (MOE for ships with freshwater ballast water source).
(DOCX)

S6 Table Potential impact of the species assessed according to known effects in invaded environments. (DOCX)

## References

[1] Stachowicz JJ, Terwin JR, Whitlatch RB, Osman RW. Linking climate change and biological invasions: ocean warming facilitates nonindigenous species invasions. Proc Natl Acad Sci. 2002;99(24):15497–500. 10.1073/pnas.242437499 12422019 PMC137745

[2] Occhipinti-Ambrogi A. Global change and marine communities: alien species and climate change. Mar Pollut Bull. 2007;55(7):342–52. 17239404 10.1016/j.marpolbul.2006.11.014

[3] Molnar JL, Gamboa RL, Revenga C, Spalding MD. Assessing the global threat of invasive species to marine biodiversity. Front Ecol Environ. 2008;6(9):485–92.

[4] Carlton JT. Transoceanic and interoceanic dispersal of coastal marine organisms: the biology of ballast water. Oceanography and Marine Biology. 1985;23:313–71.

[5] Kolar CS, Lodge DM. Ecological predictions and risk assessment for alien fishes in North America. Science. 2002;298(5596):1233–6. 10.1126/science.1075753 12424378

[6] Herborg L-M, Jerde CL, Lodge DM, Ruiz GM, MacIsaac HJ. Predicting invasion risk using measures of introduction effort and environmental niche models. Ecol Appl. 2007;17(3):663–74. 17494387 10.1890/06-0239

[7] Ruiz GM, Fofonoff PW, Carlton JT, Wonham MJ, Anson HH. Invasion of coastal marine communities in North America: Apparent patterns, processes, and biases. Annu Rev Ecol Syst. 2000;31:481–531.

[8] Coutts ADM, Dodgshun TJ. The nature and extent of organisms in vessel sea-chests: a protected mechanism for marine bioinvasions. Mar Pollut Bull. 2007;54(7):875–86. 10.1016/j.marpolbul.2007.03.011 17498747

[9] Minchin D, Gollasch S, Cohen AN, Hewitt CL, Olenin S. Characterizing vectors of marine invasion. Biological invasions in marine ecosystems: Springer; 2009 p. 109–16.

[10] Kaluza P, Kölzsch A, Gastner MT, Blasius B. The complex network of global cargo ship movements. Journal of the Royal Society Interface. 2010;7(48):1093–103. 10.1098/rsif.2009.0495 PMC2880080 20086053

[11] Xu J, Wickramarathne TL, Chawla NV, Grey EK, Steinhaeuser K;, Keller RP, et al., editors. Improving management of aquatic invasions by integrating shipping network, ecological, and environmental data: data mining for social good. In Proceedings of the 20th ACM SIGKDD international conference on Knowledge discovery and data mining. 2014. pp. 1699–1708.

[12] Thresher RE, Kuris AM. Options for managing invasive marine species. Biol Invasions. 2004;6(3):295–300.

[13] Ruiz GM, Fofonoff PW, Steves BP, Carlton JT. Invasion history and vector dynamics in coastal marine ecosystems: A North American perspective. Aquat Ecosyst Health Manage. 2015;18(3):299–311.

[14] Simard N, Plourde S, Gilbert M, Gollasch S. Net efficacy of open ocean ballast water exchange on plankton communities. J Plankton Res. 2011;33(9):1378–95.

[15] Ruiz GM, Reid DF. Current state of understanding about the effectiveness of ballast water exchange (BWE) in reducing aquatic nonindigenous species (ANS) introductions to the Great Lakes Basin and Chesapeake Bay, USA: synthesis and analysis of existing information: NOAA Tech. Memo. GLERL-142: p. xiv + 126 2007.

[16] Bailey SA, Deneau MG, Jean L, Wiley CJ, Leung B, MacIsaac HJ. Evaluating efficacy of an environmental policy to prevent biological invasions. Environ Sci Technol. 2011;45(7):2554–61. 10.1021/es102655j 21388172

[17] Cordell JR, Lawrence DJ, Ferm NC, Tear LM, Smith SS, Herwig RP. Factors influencing densities of non‐indigenous species in the ballast water of ships arriving at ports in Puget Sound, Washington, United States. Aquat Conserv: Mar Freshwat Ecosyst. 2009;19(3):322–43.

[18] Doney SC, Ruckelshaus M, Emmett Duffy J, Barry JP, Chan F, English CA, et al Climate change impacts on marine ecosystems. Ann Rev Mar Sci. 2012;4(1):11–37. 10.1146/annurev-marine-041911-111611 22457967

[19] Barnhart KR, Miller CR, Overeem I, Kay JE. Mapping the future expansion of Arctic open water. Nat Clim Chang. 2016;6(3):280–5.

[20] Meier WN, Hovelsrud GK, Oort BEH, Key JR, Kovacs KM, Michel C, et al Arctic sea ice in transformation: A review of recent observed changes and impacts on biology and human activity. Rev Geophys. 2014;52(3):185–217.

[21] Stroeve J, Serreze M, Holland M, Kay J, Malanik J, Barrett A. The Arctic’s rapidly shrinking sea ice cover: a research synthesis. Clim Change. 2012;110(3–4):1005–27.

[22] Stroeve J, Holland MM, Meier W, Scambos T, Serreze M. Arctic sea ice decline: Faster than forecast. Geophys Res Lett. 2007;34(9).

[23] Kirchmeier-Young MC, Zwiers FW, Gillett NP. Attribution of extreme events in Arctic sea ice extent. J Clim. 2017;30(2):553–71.

[24] Drake JM, Lodge DM. Global hot spots of biological invasions: Evaluating options for ballast–water management. Proceedings of the Royal Society of London B: Biological Sciences. 2004;271(1539):575–80. 10.1098/rspb.2003.2629 PMC1691629 15156914

[25] Smith LC, Stephenson SR. New Trans-Arctic shipping routes navigable by midcentury. Proc Natl Acad Sci. 2013;110(13):E1191–E5. 10.1073/pnas.1214212110 23487747 PMC3612651

[26] Miller AW, Ruiz GM. Arctic shipping and marine invaders. Nat Clim Chang. 2014;4(6):413–6.

[27] Gavrilchuk K, Lesage V. Large-scale marine development projects (mineral, oil and gas, infrastructure) proposed for Canada's North. Can Tech Rep Fish Aquat Sci. 2014.

[28] Melia N, Haines K, Hawkins E. Sea ice decline and 21st century trans‐Arctic shipping routes. Geophys Res Lett. 2016;43(18):9720–8.

[29] Pizzolato L, Howell SEL, Dawson J, Laliberté F, Copland L. The influence of declining sea ice on shipping activity in the Canadian Arctic. Geophys Res Lett. 2016;43(23):12,146–12,54.

[30] Niimi AJ. Environmental and economic factors can increase the risk of exotic species introductions to the Arctic region through increased ballast water discharge. Environ Manage. 2004;33(5):712–8. 10.1007/s00267-004-3072-4 15503388

[31] Ware C, Berge J, Sundet JH, Kirkpatrick JB, Coutts ADM, Jelmert A, et al Climate change, non-indigenous species and shipping: assessing the risk of species introduction to a high-Arctic archipelago. Divers Distrib. 2014;20(1):10–9.

[32] Goldsmit J, Archambault P, Chust G, Villarino E, Liu G, Lukovich JV, et al Projecting present and future habitat suitability of ship-mediated aquatic invasive species in the Canadian Arctic. Biol Invasions. 2018;20(2):501–17.

[33] Streftaris N, Zenetos A, Papathanassiou E. Globalisation in marine ecosystems: the story of non-indigenous marine species across European seas. Oceanogr Mar Biol Annu Rev. 2005;43:419–53.

[34] Chan FT, MacIsaac HJ, Bailey SA. Relative importance of vessel hull fouling and ballast water as transport vectors of nonindigenous species to the Canadian Arctic. Can J Fish Aquat Sci. 2015;72(8):1230–42.

[35] Chan FT, Stanislawczyk K, Sneekes AC, Dvoretsky A, Gollasch S, Minchin D, et al Climate change opens new frontiers for marine species in the Arctic: Current trends and future invasion risks. Global Change Biol. 2019;25(1):25–38. 10.1111/gcb.14469 PMC7379606 30295388

[36] Mathieson AC, Moore GE, Short FT. A floristic comparison of seaweeds from James Bay and three contiguous northeastern Canadian Arctic sites. Rhodora. 2010;112(952):396–434.

[37] Golder. (Golder Associates Ltd.) 2017 marine environmental effects monitoring program (MEEMP) and aquatic invasive species (AIS) monitoring program. Report No. 1663724-048-R-Rev0. Mary River Project; submitted to Baffinland Iron Mines Corporation, Oakville, ON. 2018. Available from http://www.baffinland.com/document-portal-new/?cat=4&archive=1&lang=en

[38] Goldsmit J, Howland KL, Archambault P. Establishing a baseline for early detection of non-indigenous species in ports of the Canadian Arctic. Aquat Invasions. 2014;9(3):327–42.

[39] Gíslason ÓS, Halldórsson HP, Pálsson MF, Pálsson S, Davíðsdóttir B, Svavarsson J. Invasion of the Atlantic rock crab (*Cancer irroratus*) at high latitudes. Biol Invasions. 2014;16(9):1865–77.

[40] Svavarsson J, Dungal P. Leyndardómar sjávarins við Ísland. Glóð, editor. Reykjavík2008.

[41] Ashton GV, Riedlecker EI, Ruiz GM. First non-native crustacean established in coastal waters of Alaska. Aquatic Biol. 2008;3(2):133–7.

[42] Lambert G, Shenkar N, Swalla BJ. First Pacific record of the north Atlantic ascidian *Molgula citrina*-bioinvasion or circumpolar distribution. Aquat Invasions. 2010;5(4):369–78.

[43] Hines AH, Ruiz GM, Fofonoff PW. Summary of NIS in Prince William Sound and Alaska. Biological invasions of cold-water coastal ecosystems: ballast-mediated introductions in Port Valdez / Prince William Sound, Alaska. Prince William Sound: Regional Citizens’ Advisory Council of Prince William Sound; 2000.

[44] Ruiz GM, Hewitt CL. Latitudinal patterns of biological invasions in marine ecosystems: a polar perspective Smithsonian Institution Scholarly Press, Washington DC 2009:347–58.

[45] Alvsvåg J, Agnalt AL, Jørstad KE. Evidence for a permanent establishment of the snow crab (*Chionoecetes opilio*) in the Barents Sea. Biol Invasions. 2009;11(3):587–95.

[46] MacDonald IR, Bluhm BA, Iken K, Gagaev S, Strong S. Benthic macrofauna and megafauna assemblages in the Arctic deep-sea Canada Basin. Deep Sea Res Part 2 Top Stud Oceanogr. 2010;57(1):136–52.

[47] Landeira JM, Matsuno K, Tanaka Y, Yamaguchi A. First record of the larvae of tanner crab *Chionoecetes bairdi* in the Chukchi Sea: A future northward expansion in the Arctic? Polar Science. 2018;16:86–9.

[48] Archambault P, Snelgrove PVR, Fisher JAD, Gagnon JM, Garbary DJ, Harvey M, et al From sea to sea: Canada's three oceans of biodiversity. PLoS ONE. 2010;5(8):e12182 10.1371/journal.pone.0012182 20824204 PMC2930843

[49] Piepenburg D, Archambault P, Ambrose WG Jr, Blanchard AL, Bluhm BA, Carroll ML, et al Towards a pan-Arctic inventory of the species diversity of the macro-and megabenthic fauna of the Arctic shelf seas. Mar Biodiv. 2011;41(1):51–70.

[50] Conservation of Arctic Flora and Fauna (CAFF). Arctic biodiversity assessment: status and trends in Arctic biodiversity Akureyri: CAFF; 2013.

[51] Force USANST. Report to the aquatic nuisance species task force. Generic nonindigenous aquatic organisms risk analysis review process (For Estimating Risk Associated with the Introduction of Nonindigenous Aquatic Organisms and how to Manage for that Risk). US Fish & Wildlife Service and National Oceanic & Atmospheric Administration. Risk Assessment and Management Committee. 1996.

[52] Hewitt CL, Hayes KR. Risk assessment of marine biological invasions Invasive aquatic species of Europe Distribution, impacts and management: Springer; 2002 p. 456–66.

[53] Ricciardi A, Rasmussen JB. Predicting the identity and impact of future biological invaders: A priority for aquatic resource management. Can J Fish Aquat Sci. 1998;55(7):1759–65.

[54] Barry SC, Hayes KR, Hewitt CL, Behrens HL, Dragsund E, Bakke SM. Ballast water risk assessment: principles, processes, and methods. ICES J Mar Sci. 2008;65(2):121–31.

[55] Kumschick S, Gaertner M, Vilà M, Essl F, Jeschke JM, Pyšek P, et al Ecological impacts of alien species: quantification, scope, caveats, and recommendations. Bioscience. 2015;65(1):55–63.

[56] Kumschick S, Richardson DM. Species‐based risk assessments for biological invasions: advances and challenges. Divers Distrib. 2013;19(9):1095–105.

[57] Moore AM, Vercaemer B, DiBacco C, Sephton D, Ma KCK. Invading Nova Scotia: first records of *Didemnum vexillum* Kott, 2002 and four more non-indigenous invertebrates in 2012 and 2013. BioInvasions Rec. 2014;3(4):225–34.

[58] Locke A. A screening procedure for potential tunicate invaders of Atlantic Canada. Aquat Invasions. 2009;4(1):71–9.

[59] Spalding MD, Fox HE, Allen GR, Davidson N, Ferdaña ZA, Finlayson MAX, et al Marine ecoregions of the world: a bioregionalization of coastal and shelf areas. Bioscience. 2007;57(7):573–83.

[60] Chan FT, Bronnenhuber JE, Bradie JN, Howland KL, Simard N, Bailey SA. Risk Assessment for ship-mediated introductions of aquatic nonindigenous species to the Canadian Arctic. Can Sci Advis Sec Res Doc. 2012.

[61] Arctic-Council. Arctic Marine Shipping Assessment. 2009. Available from: http://library.arcticportal.org/id/eprint/1400

[62] Têtu P-L, Pelletier J-F, Lasserre F. The mining industry in Canada north of the 55th parallel: a maritime traffic generator? Polar Geogr. 2015;38(2):107–22.

[63] Laget F. Transport d’espèces de dinoflagellés potentiellement non-indigènes dans l’Arctique canadien, suite au déversement des eaux de ballast par un navire domestique Rimouski: Université du Québec à Rimouski 2017.

[64] Tremblay P. Évaluation du risque potentiel d'introduction d'espèces non-indigènes de mésozooplancton suite au déversement des eaux de ballast d'un navire domestique dans l'Arctique canadien Rimouski: Univeristé du Québec à Rimouski 2017.

[65] Aarluk-Consulting, Gartner Lee Limited, Chris Anderson. Strategic plan for the Iqaluit deepwater port project. 2005. Available from: http://www.tunngavik.com/documents/publications

[66] David M, Gollasch S, Leppäkoski E, Hewitt C. Risk assessment in ballast water management Global Maritime Transport and Ballast Water Management: Springer; 2015 pp. 133–69.

[67] Bouma TJ, Olenin S, Reise K, Ysebaert T. Ecosystem engineering and biodiversity in coastal sediments: posing hypotheses. Helgol Mar Res. 2009;63(1):95–106.

[68] Murphy DJ. A comparative study of the freezing tolerances of the marine snails *Littorina littorea* (L.) and *Nassarius obsoletus* (Say). Physiol Zool. 1979;52(2):219–30.

[69] Carlton JT. Introduced marine and estuarine mollusks of North America: an end-of-the-20th-century perspective. J Shellfish Res. 1992;11(2):489–505.

[70] Chase ME, Thomas MLH. The effect of the rate and onset of temperature increase on spawning of the periwinkle, *Littorina littorea* (L.). J Exp Mar Biol Ecol. 1995;186(2):277–87.

[71] Reid DG. Systematics and evolution of *Littorina*: Ray society London; 1996.

[72] Chang AL, Blakeslee AMH, Miller AW, Ruiz GM. Establishment failure in biological invasions: a case history of *Littorina littorea* in California, USA. PLoS ONE. 2011;6(1):e16035 10.1371/journal.pone.0016035 21264336 PMC3018467

[73] Brawley SH, Coyer JA, Blakeslee AMH, Hoarau G, Johnson LE, Byers JE, et al Historical invasions of the intertidal zone of Atlantic North America associated with distinctive patterns of trade and emigration. Proc Natl Acad Sci. 2009;106(20):8239–44. 10.1073/pnas.0812300106 19416814 PMC2677092

[74] Fretter V, Graham A. The prosobranch molluscs of Britain and Denmark. J Moll Stud (Suppl. 15). 1985.

[75] Thorson G, Jørgensen CB. Reproduction and larval development of Danish marine bottom invertebrates. Medd Komm Dan Fisk Havunders Ser Plankt, 1946; 4:1–523.

[76] Morgan RP, Block SB, Ulanowicz NI, Buys C. Genetic variation in the soft-shelled clam, *Mya arenaria*. Estuaries. 1978;1(4):255–8.

[77] Goshima S. Population dynamics of the soft clam, *Mya arenaria* L., with special reference to its life history pattern. Publ Amakusa Mar Biol Lab. 1982;6:119–65.

[78] Englund VPM, Heino MP. *In situ* measurement of seasonal variation in burial depth of *Mya arenaria* Linné. J Molluscan Stud. 1994;60(4):465–7.

[79] Strasser M. *Mya arenaria*—an ancient invader of the North Sea coast. Helgoländer Meeresuntersuchungen. 1998;52(3–4):309–24.

[80] Obolewski K, Piesik Z. *Mya arenaria* (L.) in the Polish Baltic Sea Coastal (Kołobrzeg-Władysławowo). Balt Coast Zone. 2005;9:13–27.

[81] Byers JE. Marine reserves enhance abundance but not competitive impacts of a harvested nonindigenous species. Ecology. 2005;86(2):487–500.

[82] Petersen JK, Hansen JW, Laursen MB, Clausen P, Carstensen J, Conley DJ. Regime shift in a coastal marine ecosystem. Ecol Appl. 2008;18(2):497–510. 18488611 10.1890/07-0752.1

[83] Carlton JT. The Inviolate Sea? Charles Elton and Biological Invasions in the World’s Oceans. Fifty Years of Inavasion Ecology. 2011:25.

[84] Orlov YI, Ivanov BG. On the introduction of the Kamchatka king crab *Paralithodes camtschatica* (Decapoda: Anomura: Lithodidae) into the Barents Sea. Mar Biol. 1978;48(4):373–5.

[85] Rodin VE. Population biology of the king crab *Paralithodes camtschatica* Tilesius in the North Pacific Ocean Report AK-SG-90-04. University of Alaska 1989.

[86] Pavlova LV, Britayev TA, Rzhavsky AV, editors. Benthos elimination by juvenile red king crabs *Paralithodes camtschaticus* (Tilesius, 1815) in the Barents Sea coastal zone: Experimental data. Springer 2007. 10.1134/s0012496607030180 17668630

[87] Oug E, Cochrane SKJ, Sundet JH, Norling K, Nilsson HC. Effects of the invasive red king crab (*Paralithodes camtschaticus*) on soft-bottom fauna in Varangerfjorden, northern Norway. Mar Biodiv. 2011;41(3):467–79.

[88] Gibbs MT, Browman HI. Risk assessment and risk management: a primer for marine scientists. ICES J Mar Sci. 2015:fsu232.

[89] Hewitt CL, Campbell ML, Gollasch S. Alien species in aquaculture: considerations for responsible use: IUCN; 2006.

[90] Therriault TW, Herborg LM, Locke A, McKindsey CW. Risk assessment for European green crab (*Carcinus maenas*) in Canadian waters. Can Sci Advis Sec Res Doc. 2008.

[91] Mandrak NE, Cudmore B, Chapman PM. National detailed-level risk assessment guidelines: assessing the biological risk of aquatic invasive species in Canada. Can Sci Advis Sec Res Doc. 2011.

[92] Casas-Monroy O, Linley RD, Adams JK, Chan FT, Drake DAR, Bailey SA. Relative invasion risk for plankton across marine and freshwater systems: Examining efficacy of proposed international ballast water discharge standards. PLoS ONE. 2015;10(3):e0118267 10.1371/journal.pone.0118267 25763859 PMC4357441

[93] McCollin T, Shanks AM, Dunn J. Changes in zooplankton abundance and diversity after ballast water exchange in regional seas. Mar Pollut Bull. 2008;56(5):834–44. 10.1016/j.marpolbul.2008.02.004 18348891

[94] Ruiz GM, Smith G. Biological study of container vessels at the Port of Oakland. Final report. 2005.

[95] Gray DK, Johengen TH, Reid DF, MacIsaac HJ. Efficacy of open‐ocean ballast water exchange as a means of preventing invertebrate invasions between freshwater ports. Limnol Oceanogr. 2007;52(6):2386–97.

[96] Keller RP, Drake JM, Drew MB, Lodge DM. Linking environmental conditions and ship movements to estimate invasive species transport across the global shipping network. Divers Distrib. 2011;17(1):93–102.

[97] Lockwood JL, Hoopes MF, Marchetti MP. Invasion ecology: John Wiley & Sons; 2007.

[98] Wonham MJ, Walton WC, Ruiz GM, Frese AM, Galil BS. Going to the source: role of the invasion pathway in determining potential invaders. Mar Ecol Prog Ser. 2001;215(1–12).

[99] Phillips SJ, Anderson RP, Schapire RE. Maximum entropy modeling of species geographic distributions. Ecol Model. 2006;190(3):231–59.

[100] Elith J, Phillips SJ, Hastie T, Dudík M, Chee YE, Yates CJ. A statistical explanation of MaxEnt for ecologists. Divers Distrib. 2011;17(1):43–57.

[101] Mandrak NE, Cudmore B. Risk assessment: Cornerstone of an aquatic invasive species program. Aquat Ecosyst Health Manage. 2015;18(3):312–20.

[102] Simard N, Hardy M. The Laurentian Channel as an alternative ballast water exchange zone: risks, analysis and recommendations. Can Sci Advis Sec Res Doc 2004.

[103] Ricciardi A, Hoopes MF, Marchetti MP, Lockwood JL. Progress toward understanding the ecological impacts of nonnative species. Ecol Monogr. 2013;83(3):263–82.

[104] Hayes KR, Barry SC. Are there any consistent predictors of invasion success? Biol Invasions. 2008;10(4):483–506.

[105] Kenchington E, Link H, Roy V, Archambault P, Siferd T, Treble M, et al Identification of mega- and macrobenthic ecologically and biologically significant areas (EBSAs) in the Hudson Bay Complex, the western and eastern Canadian Arctic DFO, Ottawa 2011.

[106] Stewart DB, Nudds SH, Howland KL, Hannah CG, Higdon JW. An ecological and oceanographical assessment of alternate ballast water exchange zones in the Canadian eastern Arctic. Can Sci Advis Sec Res Doc. 2015.

[107] Goldsmit J, Nudds SH, Stewart DB, Higdon JW, Hannah CG, Howland KL. Where else? Assessing zones of alternate ballast water exchange in the Canadian eastern Arctic. Mar Pollut Bull. 2019;139:74–90. 10.1016/j.marpolbul.2018.11.062 30686452

[108] Chan F, Bailey S, Wiley C, MacIsaac H. Relative risk assessment for ballast-mediated invasions at Canadian Arctic ports. Biol Invasions. 2013;15(2):295–308.

[109] DiBacco C, Humphrey DB, Nasmith LE, Levings CD. Ballast water transport of non-indigenous zooplankton to Canadian ports. ICES J Mar Sci. 2012;69(3):483–91.

[110] Verling E, Ruiz GM, Smith LD, Galil B, Miller AW, Murphy KR. Supply-side invasion ecology: characterizing propagule pressure in coastal ecosystems. Proc R Soc Lond B Biol Sci. 2005;272(1569):1249–57. 10.1098/rspb.2005.3090 PMC1564104 16024389

[111] Lockwood JL, Cassey P, Blackburn T. The role of propagule pressure in explaining species invasions. Trends Ecol Evol. 2005;20(5):223–8. 10.1016/j.tree.2005.02.004 16701373

[112] Hedge LH, O'Connor WA, Johnston EL. Manipulating the intrinsic parameters of propagule pressure: implications for bio‐invasion. Ecosphere. 2012;3(6):1–13.

[113] Drake JM, Lodge DM, Lewis M. Theory and preliminary analysis of species invasions from ballast water: controlling discharge volume and location. Am Midl Nat. 2005;154(2):459–70.

[114] Carlton JT. Pattern, process, and prediction in marine invasion ecology. Biol Conserv. 1996;78(1):97–106.

[115] Ruiz GM, Carlton JT, Grosholz ED, Hines AH. Global invasions of marine and estuarine habitats by non-indigenous species: mechanisms, extent, and consequences. Am Zool. 1997;37(6):621–32.

[116] Briski E, Ghabooli S, Bailey SA, MacIsaac HJ. Invasion risk posed by macroinvertebrates transported in ships’ ballast tanks. Biol Invasions. 2012;14(9):1843–50.

[117] Johannesson K. The paradox of Rockall: why is a brooding gastropod (*Littorina saxatilis*) more widespread than one having a planktonic larval dispersal stage (*L*. *littorea*)? Mar Biol. 1988;99(4):507–13.

[118] Thorson G. Reproductive and larval ecology of marine bottom invertebrates. Biol Rev. 1950;25(1):1–45. 24537188 10.1111/j.1469-185x.1950.tb00585.x

[119] Carlton JT. Introduced species in US coastal waters PewOceans Commission, Arlington, Virginia, USA 2001.

[120] Carver CE, Mallet AL. An assessment of the risk of ballast water-mediated introduction of non-indigenous phytoplankton and zooplankton into Atlantic Canadian waters Dartmouth, NS: Transport Canada 2002.

[121] Gollasch S, Lenz J, Dammer M, Andres H-G. Survival of tropical ballast water organisms during a cruise from the Indian Ocean to the North Sea. J Plankton Res. 2000;22(5):923–37.

[122] McCollin T, Shanks AM, Dunn J. The efficiency of regional ballast water exchange: Changes in phytoplankton abundance and diversity. Harmful Algae. 2007;6(4):531–46.

[123] Renaud PE, Sejr MK, Bluhm BA, Sirenko B, Ellingsen IH. The future of Arctic benthos: expansion, invasion, and biodiversity. Prog Oceanogr. 2015;139:244–57.

[124] Vermeij GJ, Roopnarine PD. The coming Arctic invasion. Science. 2008;321(5890):780–1. 10.1126/science.1160852 18687946

[125] Bomford M. Risk assessment models for establishment of exotic vertebrates in Australia and New Zealand: Invasive Animals Cooperative Research Centre Canberra, Australia; 2008.

[126] Falk-Petersen J, Renaud P, Anisimova N. Establishment and ecosystem effects of the alien invasive red king crab (*Paralithodes camtschaticus*) in the Barents Sea–a review. ICES Jf Mar Sci. 2011;68(3):479–88.

[127] Sousa R, Gutiérrez JL, Aldridge DC. Non-indigenous invasive bivalves as ecosystem engineers. Biol Invasions. 2009;11(10):2367–85.

[128] Papacostas KJ, Rielly-Carroll EW, Georgian SE, Long DJ, Princiotta SD, Quattrini AM, et al Biological mechanisms of marine invasions. Mar Ecol Prog Ser. 2017;565:251–68.

[129] Harley CDG, Anderson KM, Lebreton CAM, MacKay A, Ayala-Díaz M, Chong SL, et al The introduction of *Littorina littorea* to British Columbia, Canada: potential impacts and the importance of biotic resistance by native predators. Mar Biol. 2013;160(7):1529–41.

[130] Drolet D, DiBacco C, Locke A, McKenzie CH, McKindsey CW, Moore AM, et al Evaluation of a new screening-level risk assessment tool applied to non-indigenous marine invertebrates in Canadian coastal waters. Biol Invasions. 2016;18(1):279–94.

[131] IMO. International Convention for the Control and Management of Ships’ Ballast Water and Sediments, 2004 (BWM 2004). Status of IMO treaties [14 May 2018], p. 506–513. 2018

[132] Jing L, Chen B, Zhang B, Peng H. A review of ballast water management practices and challenges in harsh and arctic environments. Environ Rev. 2012;20(2):83–108.

[133] Williams SL, Davidson IC, Pasari JR, Ashton GV, Carlton JT, Crafton RE, et al Managing multiple vectors for marine invasions in an increasingly connected world. Bioscience. 2013;63(12):952–66.

[134] Frey MA, Simard N, Robichaud DD, Martin JL, Therriault TW. Fouling around: vessel sea-chests as a vector for the introduction and spread of aquatic invasive species. Manag Biol Invasion. 2014;5(1):21–30.

[135] Villac MC, Kaczmarska I. Estimating propagule pressure and viability of diatoms detected in ballast tank sediments of ships arriving at Canadian ports. Mar Ecol Prog Ser. 2011;425:47–61.

[136] Casas-Monroy O, Roy S, Rochon A. Ballast sediment-mediated transport of non-indigenous species of dinoflagellates on the East Coast of Canada. Aquat Invasion. 2011;6:231–48.

[137] Villac MC, Persich G, Fernandes L, Paranhos R, Bonecker S, Garcia V, et al Ballast water exchange: testing the dilution method. Harmful Algae. 2000:470–3.

[138] García-de-Lomas J, Vilà M. Lists of harmful alien organisms: Are the national regulations adapted to the global world? Biol Invasions. 2015:1–11.

[139] Burgiel S, Foote G, Orellana M, Perrault A. Invasive alien species and trade: integrating prevention measures and international trade rules The Center for International Environmental Law and Defenders of Wildlife, Washington, DC 2006:66–74.

[140] Genovesi P, Shine C. European strategy on invasive alien species: Convention on the Conservation of European Wildlife and Habitats (Bern Convention): Council of Europe; 2011.

