# Supplementary material for: Ecological risk assessment of predicted marine invasions in the Canadian Arctic
Source: PLoS One. 2019 Feb 7;14(2):e0211815. doi: 10.1371/journal.pone.0211815 (PMC6366784; doi:10.1371/journal.pone.0211815)
Supplement: S1 Fig — (PDF) [file pone.0211815.s001.pdf]

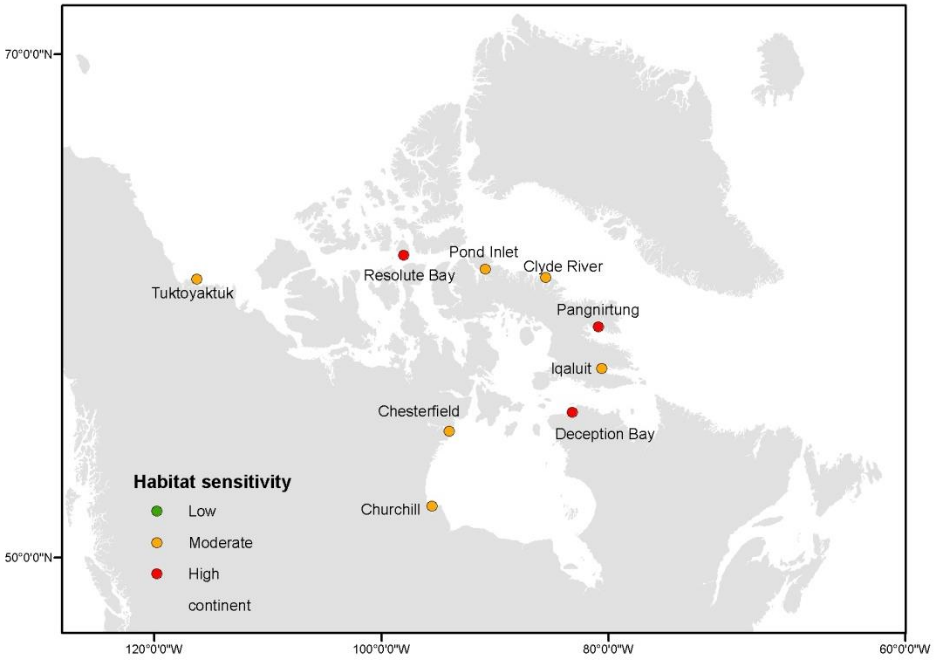

S1 Fig. Ports showing locations and habitat sensitivity according to the overlap of sensitivity variables.
