# Supplementary material for: Ecological risk assessment of predicted marine invasions in the Canadian Arctic
Source: PLoS One. 2019 Feb 7;14(2):e0211815. doi: 10.1371/journal.pone.0211815 (PMC6366784; doi:10.1371/journal.pone.0211815)
Supplement: S1 Table — Volumes are given in metric tons (MT). Correction factor for ballast water exchange: 1 (no exchange), 0.1 (mid ocean exchange (MOE), considered for ships with a saline/brackish ballast water source), 0.01 (MOE for ships with freshwater ballast water source). (DOCX) [file pone.0211815.s002.docx]

**S1 Table. Complete information on ballast water discharged at each Canadian Arctic port through domestic vessels with ballast water from regions where *Littorina littorea* is present.** Volumes are given in metric tons (MT). Correction factor for ballast water exchange: 1 (no exchange), 0.1 (mid ocean exchange (MOE), considered for ships with a saline/brackish ballast water source), 0.01 (MOE for ships with freshwater ballast water source).

| **Arrival Date** | Arrival Port (Domestic) | **Ballast water source** | **Source port** | **Total Volume / tank discharged per vessel (MT)** | **Exchange Type** | **Correction factor** | **Corrected volume (MT)** | **BW discharged at port** |
| --- | --- | --- | --- | --- | --- | --- | --- | --- |
| 29/09/2007 | Chesterfield | Sydney | Sydney | 0 | No exchange | 1 | 0 | no |
| 02/10/2005 | Churchill | Port Alfred | Port Alfred | 11397 | No exchange | 1 | 11397 | yes |
| 07/10/2005 | Churchill | Sept Iles | Sept Iles | 11397 | No exchange | 1 | 11397 | yes |
| 18/10/2005 | Churchill | Port Alfred | Port Alfred | 22210 | No exchange | 1 | 22210 | yes |
| 18/08/2007 | Churchill | Port Alfred | Port Alfred | 16074 | MOE | 0.1 | 1607.4 | yes |
| 06/08/2013 | Churchill | Port Alfred | Port Alfred | 5825.92 | No exchange | 1 | 5825.92 | yes |
| 16/08/2014 | Churchill | Port Alfred | Port Alfred | 11663.4 | No exchange | 1 | 11663.4 | yes |
| 29/09/2014 | Churchill | Sept Iles | Sept Iles | 16764.7 | MOE | 0.1 | 1676.47 | yes |
| 27/02/2005 | Deception Bay | Quebec | Quebec | 10253 | Coastal | 1 | 10253 | yes |
| 15/06/2005 | Deception Bay | Chicoutimi | Chicoutimi | 10253 | Coastal | 1 | 10253 | yes |
| 22/08/2005 | Deception Bay | Quebec | Quebec | 10253 | Coastal | 1 | 10253 | yes |
| 01/10/2005 | Deception Bay | Montreal | Montreal | 10253 | Coastal | 1 | 10253 | yes |
| 01/11/2005 | Deception Bay | Chicoutimi | Chicoutimi | 10253 | Coastal | 1 | 10253 | yes |
| 30/12/2005 | Deception Bay | Quebec | Quebec | 10253 | Coastal | 1 | 10253 | yes |
| 15/03/2006 | Deception Bay | Chicoutimi | Chicoutimi | 10253 | Coastal | 1 | 10253 | yes |
| 18/06/2006 | Deception Bay | Chicoutimi | Chicoutimi | 10253 | Coastal | 1 | 10253 | yes |
| 22/10/2006 | Deception Bay | Chicoutimi | Chicoutimi | 10253 | Coastal | 1 | 10253 | yes |
| 27/11/2006 | Deception Bay | Montreal | Montreal | 10253 | Coastal | 1 | 10253 | yes |
| 26/12/2006 | Deception Bay | Quebec | Quebec | 10253 | Coastal | 1 | 10253 | yes |
| 04/02/2007 | Deception Bay | Quebec | Quebec | 10253 | Coastal | 1 | 10253 | yes |
| 26/03/2007 | Deception Bay | Chicoutimi | Chicoutimi | 10253 | Coastal | 1 | 10253 | yes |
| 27/08/2007 | Deception Bay | Mulgrave | Mulgrave | 0 | Coastal | 1 | 0 | no |
| 13/09/2007 | Deception Bay | Lower Cove | Lower Cove | 0 | No exchange | 1 | 0 | no |
| 23/09/2007 | Deception Bay | Lower Cove | Lower Cove | 0 | No exchange | 1 | 0 | no |
| 11/11/2007 | Deception Bay | Saint John | Saint John | 3653 | MOE | 0.1 | 365.3 | yes |
| 04/01/2008 | Deception Bay | Chicoutimi | Chicoutimi | 10253 | Coastal | 1 | 10253 | yes |
| 07/03/2008 | Deception Bay | Chicoutimi | Chicoutimi | 10253 | Coastal | 1 | 10253 | yes |
| 18/04/2008 | Deception Bay | Chicoutimi | Chicoutimi | 5712 | Coastal | 1 | 5712 | yes |
| 17/06/2008 | Deception Bay | Becancour | Becancour | 10253 | Coastal | 1 | 10253 | yes |
| 22/07/2008 | Deception Bay | Montreal | Montreal | 10253 | Coastal | 1 | 10253 | yes |
| 11/08/2008 | Deception Bay | Quebec | Quebec | 10253 | Coastal | 1 | 10253 | yes |
| 28/08/2008 | Deception Bay | Belledune | Belledune | 5000 | No exchange | 1 | 5000 | yes |
| 15/09/2008 | Deception Bay | Quebec | Quebec | 10253 | Coastal | 1 | 10253 | yes |
| 11/10/2008 | Deception Bay | Chicoutimi | Chicoutimi | 10253 | Coastal | 1 | 10253 | yes |
| 19/12/2008 | Deception Bay | Chicoutimi | Chicoutimi | 10253 | Coastal | 1 | 10253 | yes |
| 15/06/2013 | Deception Bay | Quebec | Quebec | 10253 | Coastal | 1 | 10253 | yes |
| 22/06/2013 | Deception Bay | Montreal | Montreal | 3419.1 | Coastal | 1 | 3419.1 | yes |
| 20/07/2013 | Deception Bay | Quebec | Quebec | 10253 | Coastal | 1 | 10253 | yes |
| 22/08/2013 | Deception Bay | Quebec | Quebec | 10253 | Coastal | 1 | 10253 | yes |
| 02/09/2013 | Deception Bay | Quebec | Quebec | 7257 | Coastal | 1 | 7257 | yes |
| 02/10/2013 | Deception Bay | Quebec | Quebec | 10253 | Coastal | 1 | 10253 | yes |
| 14/10/2013 | Deception Bay | Summerside | Summerside | 5671 | No exchange | 1 | 5671 | yes |
| 18/10/2013 | Deception Bay | Quebec | Quebec | 4774 | Coastal | 1 | 4774 | yes |
| 03/11/2013 | Deception Bay | Quebec | Quebec | 10253 | Coastal | 1 | 10253 | yes |
| 05/12/2013 | Deception Bay | Quebec | Quebec | 10253 | Coastal | 1 | 10253 | yes |
| 11/01/2014 | Deception Bay | Quebec | Quebec | 10253 | Coastal | 1 | 10253 | yes |
| 28/02/2014 | Deception Bay | Quebec | Quebec | 10253 | Coastal | 1 | 10253 | yes |
| 15/06/2014 | Deception Bay | Quebec | Quebec | 10253 | Coastal | 1 | 10253 | yes |
| 22/07/2014 | Deception Bay | Contrecoeur | Contrecoeur | 7363 | Coastal | 1 | 7363 | yes |
| 23/08/2014 | Deception Bay | Quebec | Quebec | 9072 | Coastal | 1 | 9072 | yes |
| 18/09/2014 | Deception Bay | Quebec | Quebec | 8432 | Coastal | 1 | 8432 | yes |
| 16/10/2014 | Deception Bay | Quebec | Quebec | 7350 | Coastal | 1 | 7350 | yes |
| 25/11/2014 | Deception Bay | Quebec | Quebec | 10253 | Coastal | 1 | 10253 | yes |
| 21/08/2005 | Kuujjuaraapik (Great Whale) | Montreal | Montreal | 690 | MOE | 0.01 | 6.9 | yes |
