# Supplementary material for: Ecological risk assessment of predicted marine invasions in the Canadian Arctic
Source: PLoS One. 2019 Feb 7;14(2):e0211815. doi: 10.1371/journal.pone.0211815 (PMC6366784; doi:10.1371/journal.pone.0211815)
Supplement: S4 Table — Volumes are given in metric tons (MT). Correction factor for ballast water exchange: 1 (no exchange), 0.1 (mid ocean exchange (MOE), considered for ships with a saline/brackish ballast water source), 0.01 (MOE for ships with freshwater ballast water source). (DOCX) [file pone.0211815.s005.docx]

**S4 Table. Complete information on ballast water discharged at each Canadian Arctic port through international vessels with ballast water from regions where *Mya arenaria* is present.** Volumes are given in metric tons (MT). Correction factor for ballast water exchange: 1 (no exchange), 0.1 (mid ocean exchange (MOE), considered for ships with a saline/brackish ballast water source), 0.01 (MOE for ships with freshwater ballast water source).

| **Arrival Date** | **Arrival Port (International)** | **Ballast water source** | **Source port** | **Total Volume / tank discharged per vessel (MT)** | **Exchange Type** | **Correction factor** | **corrected volume (MT)** | **BW discharged at port** |
| --- | --- | --- | --- | --- | --- | --- | --- | --- |
| 11/08/2005 | Churchill | Newport News | Newport News | 2779 | MOE | 0.1 | 277.9 | yes |
| 03/09/2005 | Churchill | Greenore | Greenore | 9188 | MOE | 0.1 | 918.8 | yes |
| 11/09/2005 | Churchill | Newport News | Newport News | 6346 | MOE | 0.1 | 634.6 | yes |
| 15/09/2005 | Churchill | Port Everglades | Port Everglades | 9309 | MOE | 0.1 | 930.9 | yes |
| 18/09/2005 | Churchill | Baltimore | Baltimore | 10489 | MOE | 0.1 | 1048.9 | yes |
| 21/09/2005 | Churchill | Ijmuiden | Ijmuiden | 17656 | MOE | 0.1 | 1765.6 | yes |
| 28/09/2005 | Churchill | Savannah | Savannah | 958 | MOE | 0.1 | 95.8 | yes |
| 06/10/2005 | Churchill | Baltimore | Baltimore | 5505 | MOE | 0.1 | 550.5 | yes |
| 20/08/2006 | Churchill | Charleston | Charleston | 17114 | MOE | 0.1 | 1711.4 | yes |
| 04/09/2006 | Churchill | Belfast | Belfast | 4764 | MOE | 0.1 | 476.4 | yes |
| 04/09/2006 | Churchill | Foynes | Belfast | 6861 | MOE | 0.1 | 686.1 | yes |
| 04/09/2006 | Churchill | Belfast | Belfast | 931 | MOE | 0.1 | 93.1 | yes |
| 04/09/2006 | Churchill | Foynes | Foynes | 6852 | MOE | 0.1 | 685.2 | yes |
| 14/09/2006 | Churchill | Aughinish | Aughinish | 0 | No exchange | 0.1 | 0 | no |
| 27/09/2006 | Churchill | Amsterdam | Amsterdam | 32901 | MOE | 0.1 | 3290.1 | yes |
| 11/10/2006 | Churchill | Terneuzen | Terneuzen | 31358 | MOE | 0.1 | 3135.8 | yes |
| 13/10/2006 | Churchill | Dublin | Falmouth | 10087 | MOE | 0.1 | 1008.7 | yes |
| 15/10/2006 | Churchill | Dublin | Dublin | 10051 | MOE | 0.1 | 1005.1 | yes |
| 11/08/2007 | Churchill | Hamburg | Hamburg | 11397 | MOE | 0.1 | 1139.7 | yes |
| 22/08/2007 | Churchill | Klaipeda | Klaipeda | 13466 | MOE | 0.1 | 1346.6 | yes |
| 04/09/2007 | Churchill | Newport | Antwerp | 5615 | MOE | 0.1 | 561.5 | yes |
| 04/09/2007 | Churchill | Newport | Newport | 5615 | MOE | 0.1 | 561.5 | yes |
| 02/10/2007 | Churchill | Bremen | Bremen | 10697 | MOE | 0.1 | 1069.7 | yes |
| 05/10/2007 | Churchill | Londonderry | Londonderry | 14226 | MOE | 0.1 | 1422.6 | yes |
| 05/10/2007 | Churchill | Liverpool | Londonderry | 3228 | MOE | 0.1 | 322.8 | yes |
| 11/10/2007 | Churchill | Dublin | Dublin | 6818 | MOE | 0.1 | 681.8 | yes |
| 11/10/2007 | Churchill | Portbury | Portbury | 9629 | MOE | 0.1 | 962.9 | yes |
| 11/10/2007 | Churchill | Dublin | Portbury | 6818 | MOE | 0.1 | 681.8 | yes |
| 16/10/2007 | Churchill | Tyne | Muuga-Port Of Tallinn | 0 | MOE | 0.1 | 0 | no |
| 08/11/2007 | Churchill | Hamburg | Unknown | 11397 | MOE | 0.1 | 1139.7 | yes |
| 04/08/2008 | Churchill | Szczecin | Szczecin | 27426 | MOE | 0.1 | 2742.6 | yes |
| 08/08/2008 | Churchill | Kaliningrad | Kaliningrad | 0 | MOE | 0.1 | 0 | no |
| 13/08/2008 | Churchill | Riga | Copenhagen | 11627 | MOE | 0.1 | 1162.7 | yes |
| 13/08/2008 | Churchill | Riga | Riga | 11624 | MOE | 0.1 | 1162.4 | yes |
| 21/08/2008 | Churchill | Gdynia | Gdynia | 7825 | MOE | 0.1 | 782.5 | yes |
| 08/09/2008 | Churchill | Teesport | Teesport | 977 | MOE | 0.1 | 97.7 | yes |
| 08/09/2008 | Churchill | Teesport | Teesport | 9188 | MOE | 0.1 | 918.8 | yes |
| 14/09/2008 | Churchill | Amsterdam | Amsterdam | 11394 | MOE | 0.1 | 1139.4 | yes |
| 22/09/2008 | Churchill | Straumsvik | Straumsvik | 11866 | MOE | 0.1 | 1186.6 | yes |
| 30/09/2008 | Churchill | Liepaja | Liepaja | 372 | MOE | 0.1 | 37.2 | yes |
| 04/10/2008 | Churchill | Ravenna | Ravenna | 11571 | MOE | 0.1 | 1157.1 | yes |
| 13/10/2008 | Churchill | Porto Marghera | Gibraltar | 8561 | MOE | 0.1 | 856.1 | yes |
| 11/08/2009 | Churchill | Liepaja | Liepaja | 9208 | MOE | 0.1 | 920.8 | yes |
| 25/08/2009 | Churchill | Brest | Portland | 5990 | MOE | 0.1 | 599 | yes |
| 30/08/2009 | Churchill | Straumsvik | Straumsvik | 10239 | MOE | 0.1 | 1023.9 | yes |
| 30/08/2009 | Churchill | Straumsvik | Straumsvik | 1888 | MOE | 0.1 | 188.8 | yes |
| 12/09/2009 | Churchill | Brunsbüttel | Ronnskar | 4338 | MOE | 0.1 | 433.8 | yes |
| 09/10/2009 | Churchill | Vlissingen | Vlissingen | 13634 | MOE | 0.1 | 1363.4 | yes |
| 27/07/2010 | Churchill | Bari | Gibraltar | 6532 | MOE | 0.1 | 653.2 | yes |
| 03/08/2010 | Churchill | Hamburg | Nuuk | 0 | MOE | 0.1 | 0 | no |
| 05/08/2010 | Churchill | Klaipeda | Klaipeda | 10570 | MOE | 0.1 | 1057 | yes |
| 17/08/2010 | Churchill | Straumsvik | Straumsvik | 14844 | MOE | 0.1 | 1484.4 | yes |
| 17/08/2010 | Churchill | Reydarfjordur | Straumsvik | 10858 | MOE | 0.1 | 1085.8 | yes |
| 24/08/2010 | Churchill | Newport | Falmouth | 9548 | MOE | 0.1 | 954.8 | yes |
| 24/08/2010 | Churchill | Falmouth | Falmouth | 880 | MOE | 0.1 | 88 | yes |
| 30/08/2010 | Churchill | Vlissingen | Vlissingen | 7500 | MOE | 0.1 | 750 | yes |
| 22/09/2013 | Churchill | Reydarfjordur | Reydarfjordur | 16071.6 | MOE | 0.1 | 1607.2 | yes |
| 27/09/2013 | Churchill | Straumsvik | Straumsvik | 10708 | MOE | 0.1 | 1070.8 | yes |
| 06/10/2013 | Churchill | Reydarfjordur | Reydarfjordur | 13092 | MOE | 0.1 | 1309.2 | yes |
| 31/10/2013 | Churchill | Karmoy | Karmoy | 10285 | MOE | 0.1 | 1028.5 | yes |
| 09/08/2014 | Churchill | Dunkirk | Dunkirk | 14713.9 | MOE | 0.1 | 1471.4 | yes |
| 31/08/2014 | Churchill | Tyne | Tyne | 14358.6 | MOE | 0.01 | 143.6 | yes |
| 25/09/2014 | Churchill | Straumsvik | Straumsvik | 13482 | MOE | 0.1 | 1348.2 | yes |
| 23/10/2014 | Churchill | Brunsbüttel | Brunsbüttel | 8840 | MOE | 0.1 | 884 | yes |
| 14/08/2010 | Clyde River | Reykjavik | Sisimiut | 0 | No exchange | 0.1 | 0 | no |
| 12/11/2007 | Deception Bay | Aahrus | Aahrus | 14946 | MOE | 0.1 | 1494.6 | yes |
| 01/10/2008 | Deception Bay | Eemshaven | Eemshaven | 0 | No exchange | 0.1 | 0 | no |
| 09/09/2010 | Deception Bay | Eemshaven | Eemshaven | 0 | MOE | 0.1 | 0 | no |
|  |  |  |  |  |  |  |  |  |
| 17/09/2011 | Deception Bay | Liverpool | Eemshaven | 234 | MOE | 0.1 | 23.4 | yes |
| 09/12/2007 | Iqaluit | Las Palmas / Shelburne | Sisimiut | 0 | No exchange | 0.1 | 0 | no |
| 11/08/2008 | Iqaluit | Reykjavik | Reykjavik | 0 | No exchange | 0.1 | 0 | no |
| 10/09/2008 | Iqaluit | Reykjavik | Reykjavik | 0 | No exchange | 0.1 | 0 | no |
| 17/09/2009 | Iqaluit | Falmouth | Qaqortoq | 0 | MOE | 0.1 | 0 | no |
| 21/08/2010 | Iqaluit | Skagen | Ventspils | 0 | MOE | 0.1 | 0 | no |
| 17/09/2010 | Iqaluit | Husavik | Narsaq | 0 | MOE | 0.1 | 0 | no |
| 20/07/2009 | Pangnirtung | Everett | Everett | 0 | No exchange | 0.1 | 0 | no |
| 22/08/2010 | Pond Inlet | Hamburg | Uummannaq | 0 | No exchange | 0.1 | 0 | no |
| 24/08/2008 | Resolute Bay (Quassuittuq) | Copenhagen | Copenhagen | 0 | No exchange | 0.1 | 0 | no |
| 19/08/2008 | Tuktoyaktuk | Ulsan | Ulsan | 0 | MOE | 0.1 | 0 | no |
| 20/08/2008 | Tuktoyaktuk | Hakodate | Hakodate | 0 | No exchange | 0.1 | 0 | no |
| 24/09/2008 | Tuktoyaktuk | Dutch Harbor | Dutch Harbor | 0 | No exchange | 0.1 | 0 | no |
| 12/08/2009 | Tuktoyaktuk | San Francisco | San Francisco | 0 | MOE | 0.1 | 0 | no |
