# Supplementary material for: Ecological risk assessment of predicted marine invasions in the Canadian Arctic
Source: PLoS One. 2019 Feb 7;14(2):e0211815. doi: 10.1371/journal.pone.0211815 (PMC6366784; doi:10.1371/journal.pone.0211815)
Supplement: S5 Table — Volumes are given in metric tons (MT). Correction factor for ballast water exchange: 1 (no exchange), 0.1 (mid ocean exchange (MOE), considered for ships with a saline/brackish ballast water source), 0.01 (MOE for ships with freshwater ballast water source). (DOCX) [file pone.0211815.s006.docx]

**S5 Table. Complete information on ballast water discharged at each Canadian Arctic port through international vessels with ballast water from regions where *Paralithodes camtschaticus* is present.** Volumes are given in metric tons (MT). Correction factor for ballast water exchange: 1 (no exchange), 0.1 (mid ocean exchange (MOE), considered for ships with a saline/brackish ballast water source), 0.01 (MOE for ships with freshwater ballast water source).

| **Arrival Date** | **Arrival Port (International)** | **Ballast water source** | **Source port** | **Total Volume / tank discharged per vessel (MT)** | **Exchange Type** | **Correction factor** | **corrected volume (MT)** | **BW discharged at port** |
| --- | --- | --- | --- | --- | --- | --- | --- | --- |
| 24/09/2008 | Tuktoyaktuk | Dutch Harbor | Dutch Harbor | 0 | No exchange | 0.1 | 0 | 0 |
