# Supplementary material for: Ecological risk assessment of predicted marine invasions in the Canadian Arctic
Source: PLoS One. 2019 Feb 7;14(2):e0211815. doi: 10.1371/journal.pone.0211815 (PMC6366784; doi:10.1371/journal.pone.0211815)
Supplement: S6 Table — (DOCX) [file pone.0211815.s007.docx]

**S6 Table. Potential impact of the species assessed according to known effects in invaded environments.**

| **Species** | **Biodiversity, abundance and distribution** | **Interspecific interactions** | **Habitat** | **Trophic interactions** | **References** |
| --- | --- | --- | --- | --- | --- |
| ***Littorina littorea*** | **Moderate** | **High** | **High** | **Moderate** | [1-8] |
|  | -Changes in abundance of natives gastropods  -Changes in abundance and biodiversity of plants and animals | -Resource competition  -Displace of native species  -Niche shifts in native gastropods | -Alters distribution and abundance of algae, converting soft sediment to hard substrates  -Changes in habitat physical conditions  -Changes in rocky intertidal community structure | -Grazing activity can change the intertidal ecosystem |  |
| ***Mya arenaria*** | **Moderate** | **Moderate** | **High** | **Moderate** | [9-11] |
|  | -Changes in phytoplankton composition and zooplankton abundance  -Reduction in biomass and coverage of benthic vegetation | -Outcompetes with native bivalves | -Affects composition and granulometric structure of shallow water and sea shore deposits  -Its shells form a secondary hard substrate available for associated species in mobile bottoms  -Change in regime shift: from pelagic turnover to benthic pelagic coupling  -Changes in salinity  -Changes in water transparency, increasing plant coverage | -Changes in benthic algae can affect herbivorous seabirds |  |
| ***Paralithodes camtschaticus*** | **High** | **High** | **High** | **High** | [12-24] |
|  | -Changes in biodiversity, reduced benthic biomass and diversity  -It can eliminate up to 15% of the coastal population of sea urchin  -Reduction in soft-bottom communities in number of large individuals. Starts to dominate small individuals | -Competition with fish such as haddock, cod, wolffish and Atlantic cod (overlap in diet) | -Modify bottom communities  -Changes in physical appearance in benthic communities and alteration of community structure  -The crabs are also physical structures themselves and may represent new habitats that could allow increased biodiversity (13 different species found as fouling on crabs carapace)  -Reduce stability of local habitats through burrowing activity | -It is a large general predator. It can predate on 100 different species (invertebrates, algae and fish remnants):  -Impact on bottom native communities  -Affect native population of scallops, eggs of lumpsucker, sea urchins, capelins through direct predation |  |

**References**

1. Brenchley GA, Carlton JT. Competitive displacement of native mud snails by introduced periwinkles in the New England intertidal zone. The Biological Bulletin. 1983;165(3):543-58.

2. Bertness MD. Habitat and community modification by an introduced herbivorous snail. Ecology. 1984:370-81.

3. Petraitis PS. Factors organizing rocky intertidal communities of New England: herbivory and predation in sheltered bays. J Exp Mar Biol Ecol. 1987;109(2):117-36.

4. Carlton JT. Molluscan invasions in marine and estuarine communities. Malacologia. 1999;41(2):439-54.

5. Eastwood MM, Donahue MJ, Fowler AE. Reconstructing past biological invasions: niche shifts in response to invasive predators and competitors. Biol Invasions. 2007;9(4):397-407.

6. Tyrrell MC, Dionne M, Edgerly JA. Physical factors mediate effects of grazing by a non-indigenous snail species on saltmarsh cordgrass (*Spartina alterniflora*) in New England marshes. ICES Journal of Marine Science: Journal du Conseil. 2008;65(5):746-52.

7. Chang AL, Blakeslee AMH, Miller AW, Ruiz GM. Establishment failure in biological invasions: a case history of *Littorina littorea* in California, USA. PLoS ONE. 2011;6(1):e16035.

8. Harley CDG, Anderson KM, Lebreton CAM, MacKay A, Ayala-Díaz M, Chong SL, et al. The introduction of *Littorina littorea* to British Columbia, Canada: potential impacts and the importance of biotic resistance by native predators. Mar Biol. 2013;160(7):1529-41.

9. Leppäkoski EJ. Introduced species—resource or threat in brackish-water seas? Examples from the Baltic and the Black Sea. Mar Pollut Bull. 1991;23:219-23.

10. Petersen JK, Hansen JW, Laursen MB, Clausen P, Carstensen J, Conley DJ. Regime shift in a coastal marine ecosystem. Ecol Appl. 2008;18(2):497-510.

11. Crocetta F, Turolla E. *Mya arenaria* Linné, 1758 (Mollusca: Bivalvia) in the Mediterranean Sea: its distribution revisited. Journal of Biological Research-Thessaloniki. 2011;16:188-93.

12. Orlov YI, Ivanov BG. On the introduction of the Kamchatka king crab *Paralithodes camtschatica* (Decapoda: Anomura: Lithodidae) into the Barents Sea. Mar Biol. 1978;48(4):373-5.

13. Tilman D. The ecological consequences of changes in biodiversity: A search for general principles 101. Ecology. 1999;80(5):1455-74.

14. Veldhuizen TC, Stanish S. Overview of the life history, distribution, abundance, and impact of the Chinese mitten crab *Eriocheir sinensis*. 1999.

15. Gudimov AV, Gudimova EN, Pavlova LV, editors. Effect of the red king crab *Paralithodes camtschaticus* on the Murmansk coastal macrobenthos: the first estimates using sea urchins of the genus *Strongylocentrotus* as an example2003 2003: Springer.

16. Haugan TA. Bunnsamfunn og næringsvalg hos konge krabbe, *Paralithodes camtschaticus* (Tilesius, 1815), på noen lokaliteter in Finnmark. Master scient-oppgave Norges Fiskerihøgskole, Universitetet i Tromsø. 2004.

17. Pavlova LV, Kuzmin SA, Rzhavsky AV, Britayev TA. On the biology and feeding of the juvenile red king crab *Paralithodes camtschaticus* from Dal'nezelenetskaya Bay (Barents Sea). Shelf zoobenthos Investigations Information support of the ecosystem investigations GG Matishov (Ed) KSC Press, Apatity, Russia. 2004:49-95.

18. Anisimova N, Berenboim B, Gerasimova O, Manushin I, Pinchukov M. On the effect of red king crab on some components of the Barents Sea ecosystem. Report PINRO, Murmansk. 2005.

19. Jørgensen LL. Impact scenario for an introduced decapod on Arctic epibenthic communities. Biol Invasions. 2005;7(6):949-57.

20. Gilbey V, Attrill MJ, Coleman RA. Juvenile Chinese mitten crabs (*Eriocheir sinensis*) in the Thames estuary: distribution, movement and possible interactions with the native crab *Carcinus maenas*. Biol Invasions. 2008;10(1):67-77.

21. Dvoretsky AG, Dvoretsky VG. Fouling community of the red king crab, *Paralithodes camtschaticus* (Tilesius 1815), in a subarctic fjord of the Barents Sea. Polar Biol. 2009;32(7):1047-54.

22. Britayev TA, Rzhavsky AV, Pavlova LV, Dvoretskij AG. Studies on impact of the alien Red King Crab (*Paralithodes camtschaticus*) on the shallow water benthic communities of the Barents Sea. J Appl Ichthyol. 2010;26(s2):66-73.

23. Falk-Petersen J, Renaud P, Anisimova N. Establishment and ecosystem effects of the alien invasive red king crab (*Paralithodes camtschaticus*) in the Barents Sea–a review. ICES Journal of Marine Science: Journal du Conseil. 2011;68(3):479-88.

24. Oug E, Cochrane SKJ, Sundet JH, Norling K, Nilsson HC. Effects of the invasive red king crab (*Paralithodes camtschaticus*) on soft-bottom fauna in Varangerfjorden, northern Norway. Mar Biodiv. 2011;41(3):467-79.
